# New *Drosophila* promoter-associated architectural protein Mzfp1 interacts with CP190 and is required for housekeeping gene expression and insulator activity

**DOI:** 10.1093/nar/gkae393

**Published:** 2024-05-20

**Authors:** Vladimir Sokolov, Olga Kyrchanova, Natalia Klimenko, Anna Fedotova, Airat Ibragimov, Oksana Maksimenko, Pavel Georgiev

**Affiliations:** Department of the Control of Genetic Processes, Institute of Gene Biology, Russian Academy of Sciences, Moscow 119334, Russia; Department of the Control of Genetic Processes, Institute of Gene Biology, Russian Academy of Sciences, Moscow 119334, Russia; Center for Precision Genome Editing and Genetic Technologies for Biomedicine, Institute of Gene Biology, Russian Academy of Sciences, Moscow 119334, Russia; Center for Precision Genome Editing and Genetic Technologies for Biomedicine, Institute of Gene Biology, Russian Academy of Sciences, Moscow 119334, Russia; Department of the Control of Genetic Processes, Institute of Gene Biology, Russian Academy of Sciences, Moscow 119334, Russia; Center for Precision Genome Editing and Genetic Technologies for Biomedicine, Institute of Gene Biology, Russian Academy of Sciences, Moscow 119334, Russia; Center for Precision Genome Editing and Genetic Technologies for Biomedicine, Institute of Gene Biology, Russian Academy of Sciences, Moscow 119334, Russia; Department of the Control of Genetic Processes, Institute of Gene Biology, Russian Academy of Sciences, Moscow 119334, Russia

## Abstract

In *Drosophila*, a group of zinc finger architectural proteins recruits the CP190 protein to the chromatin, an interaction that is essential for the functional activity of promoters and insulators. In this study, we describe a new architectural C2H2 protein called Madf and Zinc-Finger Protein 1 (Mzfp1) that interacts with CP190. Mzfp1 has an unusual structure that includes six C2H2 domains organized in a C-terminal cluster and two tandem MADF domains. Mzfp1 predominantly binds to housekeeping gene promoters located in both euchromatin and heterochromatin genome regions. *In vivo* mutagenesis studies showed that Mzfp1 is an essential protein, and both MADF domains and the CP190 interaction region are required for its functional activity. The C2H2 cluster is sufficient for the specific binding of Mzfp1 to regulatory elements, while the second MADF domain is required for Mzfp1 recruitment to heterochromatin. Mzfp1 binds to the proximal part of the *Fub* boundary that separates regulatory domains of the *Ubx* and *abd-A* genes in the *Bithorax* complex. Mzfp1 participates in *Fub* functions in cooperation with the architectural proteins Pita and Su(Hw). Thus, Mzfp1 is a new architectural C2H2 protein involved in the organization of active promoters and insulators in *Drosophila*.

## Graphical Abstract

**Graphical Abstract:**
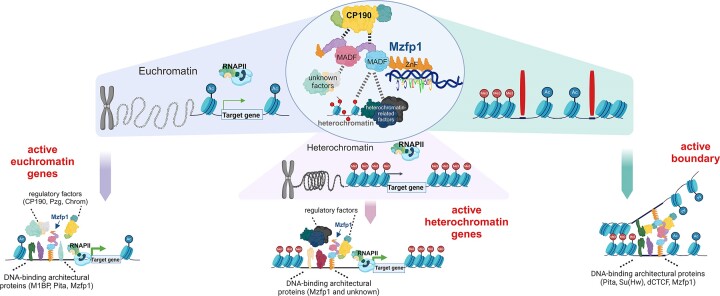

## Introduction

Gene expression in higher eukaryotes is determined by the interaction of enhancers and promoters, a process that can form regulatory hubs (1–6). Housekeeping genes that are ubiquitously expressed often have enhancers or upstream regulatory regions located in close proximity to the promoters (7,8). In contrast, developmentally regulated genes are controlled by many independent enhancers that are frequently located far from the promoters (9,10). Enhancers determine the timing and cell-specific activation of gene expression. The core promoter serves as a scaffold for the assembly of a pre-initiation complex (PIC) composed of basal transcription factors (1,11). TFIID, a key basal transcription factor that recognizes promoters, comprises a TATA box-binding protein (TBP) along with 13–14 TBP-associated factors (TAFs). TBPs and some TAFs bind to specific core promoter motifs. However, in higher eukaryotes, only a minor proportion of promoters contain such motifs, raising the question of how the main transcription factors recognize and bind promoter sequences (3,11). This question becomes even more intriguing since it was found that a significant portion of active genes in *Drosophila* are located in constitutive heterochromatin (12,13).

In *Drosophila*, several architectural proteins have been characterized that organize and support long-distance interactions in chromosomes (10,14,15). These proteins belong to a large group characterized by a similar organization of the DNA-binding domain that consists of an array of properly spaced zinc-finger domains of the C2H2 type (C2H2 proteins) (16,17). C2H2 proteins specifically bind to long DNA motifs (18–21), distinguishing this class of proteins from other transcription factors that usually recognize short and degenerate motifs (21–26). Architectural C2H2 proteins usually contain homodimerization domains at the N-termini that are essential for organizing distant interactions (16). The most conserved architectural C2H2 protein among higher eukaryotes, CTCF, contains an unstructured N-terminal region that can homodimerize (27,28). *Drosophila* Pita, Zw5, ZIPIC, and ZAF1 proteins have an N-terminal zinc finger-associated domain (ZAD) that predominantly forms homodimers and is critical for supporting specific distant interactions with architectural proteins (29–32).

Despite the fact that the known C2H2 architectural proteins predominantly bind to gene promoters, the extent of their role in promoter organization remains underestimated (31–37). For example, the ZAD-C2H2 protein, named Motif 1 Binding Protein (M1BP), predominantly binds to the promoters of at least 2000 active genes (38). Experimental data suggest that M1BP is involved in chromatin opening and that it helps recruit main transcription factors to promoters (39,40). It has also been demonstrated that the expression of the *neverland* and *spookier* genes located in constitutive heterochromatin depends on three ZAD-C2H2 proteins, Sean, Ouib and Mld, binding to their promoters (41).

According to the current model (11,14,16), architectural proteins can support open chromatin in promoter regions and participate in the recruitment of basal transcription factors. The primary binding partner of many characterized architectural proteins is CP190, a protein that belongs to a large family of transcription factors containing an N-terminal BTB (Broad-Complex, Tramtrack, and Bric-à-brac) domain (40,42,43). CP190 is involved in the formation of an open chromatin structure (44–46) and the recruitment of the nucleosome remodeling factor (NURF), the dimerization partner, RB-like, E2F and multi-vulval class B (dREAM), and the Spt-Ada-Gcn5 acetyltransferase (SAGA) complexes (47–51). Several studies have suggested that CP190 is involved in long-distance interactions between regulatory elements as well as in insulator activity and chromosome architecture (46,52–55).

The architectural proteins Su(Hw), dCTCF and Pita form boundaries in the *Bithorax* complex (BX-C) that contains three homeotic genes, *Ultrabithorax* (*Ubx*), *abdominal-A* (*abd-A*) and *Abdominal-B* (*Abd-B*) (35,56–58). The expression of each homeotic gene in the appropriate parasegment-specific pattern is controlled by a series of nine *cis*-regulatory domains, *abx/bx*, *bxd/pbx* and *iab-2* to *iab-9* (59–61). The boundaries are required for the functional autonomy of the regulatory domains, and they support enhancer-promoter communication (56,62–68). *Fub* (*Fab-2*), *Mcp*, *Fab-6*, *Fab-7* and *Fab-8* are the most thoroughly characterized BX-C boundaries that contain different combinations of Pita, Su(Hw) and dCTCF sites (56,58,62,63,65,66,69). However, additional unknown architectural proteins are required for the activity of the BX-C boundaries (58). The Pita, Su(Hw), and dCTCF proteins are responsible for recruiting CP190 to the boundaries involved in insulator functions (46,58,70,71).

Here, we characterized a new C2H2 protein (CG1603) that contains a cluster of six C2H2 zinc-finger domains at the C-terminus and two MADF domains in the central region. We named this protein Madf and Zinc-Finger Protein 1 (Mzfp1) due to these features. The MADF (myb/SANT-like domain in Adf-1) domain is an approximately 80-amino-acid module that is present in a large group of transcription factors in *Drosophila* (72–74). The first characterized factor Adf-1, which binds to promoters and is required for transcription stimulation (75), was shown to have its N-terminal MADF domain bind to a site consisting of multiple trinucleotide repeats (73,75). However, the MADF domain is also essential for transcriptional activation by Adf-1, suggesting that it is involved in recruiting complexes required for promoter stimulation (76). In contrast, the MADF-containing protein Stonewall (Stwl) has an opposite function in organizing the heterochromatin (77).

Our findings indicate that Mzfp1 interacts with CP190 and primarily binds to the promoters of housekeeping genes in both euchromatin and heterochromatin. Mutations of the *mzfp1* gene were lethal, indicating that Mzfp1 is important during *Drosophila* development. The second MADF domain is critical for the association of Mzfp1 with heterochromatin. Additionally, Mzfp1 binds to the *Fub* boundary, thereby blocking the crosstalk between the *bxd/pbx* and *iab-2* domains that regulate the *Ubx* and *abd-A* genes of BX-C. Our results also suggest that Mzfp1 cooperates with Pita and Su(Hw) in organizing the *Fub* boundary.

## Materials and methods

### Generation and validation of fly lines with deletion of the *CG1603^attP^* gene using CRISPR/Cas9

The *CG1603^attP^* deletion was obtained by CRISPR/Cas9-induced homologous recombination (78). The guide RNAs were selected using the program ‘CRISPR optimal target finder’ (http://targetfinder.flycrispr.neuro.brown.edu/, O’Connor-Giles Lab): the 5′ target site was selected in the gene's *5’UTR*, the 3′ target site was selected in the *3’UTR* (Supplementary Table S1). The 5′ and 3′ targets were cloned into the pCR vector based on *pCFD4-U6:1_U6:3tandemgRNAs* plasmid (Addgene # 49411), using *BbsI*. As a reporter, we used *pHD-DsRed* vector that was a gift from Kate O’Connor-Giles (Addgene plasmid # 51434). The plasmid was constructed in the following order: *proximal arm-attP-loxP*-*3×Р3:DsRed-loxP-distal arm*. Arms for homology recombination were amplified by PCR from DNA isolated from *Oregon* line. The breakpoints of the designed deletion: 2R:7516276..7514199 according r6.36. To generate the desired deletions, the plasmid construct (300 ng/μl) was injected into *58492* line (Bloomington *Drosophila* Stock Center) embryos together with gRNA expressing vector. Flies with potential deletions were selected on the basis of dsRed signal in eyes and these flies were crossed with *y^1^w^1118^*; *CyO* balancer line. All independently obtained flies with *dsRed* reporter were tested by PCR.

### Generation of transgenic fly lines and rescuing of the *CG1603^attP^* mutation

To express 3×HA-tagged Mfzp1 and it's deletion derivatives, protein-coding sequences of Mfzp1 were cloned in frame with 3×HA and linker (A(EAAAK)_2_A, as described in (79)). PCR directed mutagenesis was used to make constructs with deletion variants of Mzfp1. Corresponding primers and linker sequence are listed in Supplementary Table S1. Deletion derivative ΔZF was cloned by excising ScaI–SalI fragment from Mfzp1-linker-3×HA construction described above. Different full-sized variants of Mzfp1 were cloned into an expression vector containing *attB* site for φC31-mediated recombination, *Ubi-p63E* promoter with its 5’UTR, 3’UTR with SV40 polyadenylation signal, intronless *yellow* gene as a reporter for detection of transformants (80).

*Drosophila* strains were grown at 25°C and 75% humidity under standard culture conditions. The transgenic constructs (U:Mzfp1*) were injected into preblastoderm embryos using the φC31-mediated site-specific integration system at locus 86F8 (line y[1] M{RFP[3xP3.PB] GFP[E.3xP3]=vas-int.Dm}ZH-2A w[*]; M{3xP3-RFP.attP}ZH-86Fb (#24749, Bloomington *Drosophila* Stock Center)) (81). F0 adults were crossed with *y^1^w^1118^* flies, and the progeny carrying the U:Mzfp1* transgene in the 86F8 region were identified by *y^+^* pigmented cuticle. Next, y^1^w^1118^; U:Mzfp1* males were crossed with *y^1^w^1118^; CyO,GFP/+; TM6,Tb*/+ females, where *CyO,GFP* is obtained from the w[1118]; sna[Sco]/CyO, P[ActGFP.w-]CC2 line (#9325, Bloomington *Drosophila* Stock Center). Obtained in progeny y^1^w^1118^; CyO,GFP/+; TM6,Tb/ U:Mzfp1* males were crossed again with *y^1^w^1118^; CG1603^attP^/ CyO; TM6,Tb*/+ females. In the next generation, males and females with y^1^w^1118^; CyO,GFP/ CG1603^attP^; TM6,Tb/ U:Mzfp1*genotype were individually crossed. Several transgenic lines with the same genotype y^1^w^1118^; CyO,GFP/ CG1603^attP^; TM6,Tb/ U:Mzfp1*were independently maintained. All stocks are available upon request.

### Electrophoretic mobility shift assay (EMSA)

To express proteins for EMSA protein-coding sequences of full-length Mzfp1, Mzfp1 zinc fingers and Su(Hw) were cloned in pMAL-c5x vector in frame with N-terminal maltose-binding protein (MBP) (primers are listed in Supplementary Table S1). Pita zinc fingers cloning was described previously (33). Recombinant proteins were expressed in *E. coli* BL21 cells and purified using standard procedures. Briefly, the cells were disrupted by sonication in basic buffer (20 mM HEPES-KOH, pH 7.6; 150 mM NaCl, 10 mM MgCl_2_, 1 mM DTT, 0.1 mM ZnSO_4_, 10% glycerol, 0.1% NP-40, 1 mM PMSF, 1:1000 Complete Protease Inhibitor Cocktail VII (Calbiochem)). The lysate was cleared by centrifugation and applied onto immobilized amylose (New England Biolabs) column. After washing, the bound proteins were eluted with maltose-containing buffer (20 mM Tris-HCl, pH 7.6; 200 mM NaCl, 0.1 mM ZnCl_2_, 10 mM maltose, 1 mM DTT). Aliquots of purified recombinant proteins were incubated with fluorescently labeled DNA fragments in the presence of nonspecific binding competitor poly(dI-dC) (0.005 pg/μl). Incubation was performed in PBS (pH 8.0) containing 5 mM MgCl_2_, 0.1 mM ZnSO_4_, 1 mM DTT, 0.1% NP-40 and 10% glycerol at room temperature for 30 min. The mixtures were then resolved by nondenaturing 5% PAGE (79 AA:1 BAA) in 0.5 × TBE buffer. Fluorescence was detected using the Chemidoc MP System (Bio-Rad, USA) at 500 nm excitation and 535 nm emission (FAM-labeled fragments), 630 nm excitation and 700 nm emission (Cy5-labeled fragments).

### Yeast two-hybrid assay

The yeast two-hybrid assay was performed as previously described (82). Fragments of *mzfp1* cDNA were fused to activation domain of GAL4, fragments of *CP190* cDNA – to DNA-binding domain of GAL4 using pGAD424 and pGBT9 vectors (Clontech, USA), respectively, with the primers listed in Supplementary Table S1. Generated plasmids were transformed into *Saccharomyces cerevisiae PJ69-4A* strain (*MATa trp1–901 leu2–3,112 ura3–52 his3–200 gal4Δ gal80Δ LYS2::GAL1-HIS3 GAL2-ADE2 met2::GAL7-lacZ*) using the LiAc/ssDNA/PEG method (83). The cells were plated on medium lacking tryptophan and leucine. After three-day growth at 30ºC, the samples were streaked on selective medium lacking tryptophan, leucine, histidine, and adenine and incubated at 30ºC. Colony growth was assessed 3 days later. Each assay was repeated three times.

### Cell culture, transfection, dual luciferase assay

*Drosophila* S2 cells were grown in SFX medium (HyClone, USA) at 25°C. Transfection of plasmids was performed with the Cellfectin II reagent (Thermo Fisher Scientific, USA) according to the manufacturer's instructions. Typically, cells were transfected in 6- or 12-well plates and grown for 24 to 48 h before harvesting. All transfection procedures were performed with three independent replicates.

The dual luciferase assay was performed with the Firefly & Renilla Luciferase Assay Kit (Biotium, USA) following the manufacturer's instructions. Luciferase activity was estimated with a Sirius 2 luminometer (Berthold Technologies, Germany). Analysis was performed in three independent biological replicates. The significance of changes in the expression level was estimated by two-sided independent Student's *t*-test without equal variance assumption.

### Co-immunoprecipitation assay

*Drosophila* S2 cells were co-transfected by 3xHA-Mzfp1 (wild-type and with deletion of CP190-interacting region) and CP190 plasmids with Cellfectin II (Thermo Fisher Scientific, USA). After transfection, the cells were incubated for 48 h and then collected by centrifugation at 700 *g* for 5 min, washed once with 1×PBS, and resuspended in 20 packed cell volumes of hypotonic lysis buffer (20 mM Tris–HCl, pH 7.4, with 10 mM KCl, 10 mM MgCl_2_, 2 mM EDTA, 10% glycerol, 1% Triton X-100, 1 mM DTT and 1:100 Calbiochem Complete Protease Inhibitor Cocktail V). After incubation on ice for 10 min, the cells were sonicated (2 × 15 s on ice at 20% output, 40% duty cycle), NaCl was added to a final concentration of 420 mM, and incubation on ice continued for 60 min, with periodic mixing. Sonication was repeated as above to reduce viscosity, cell debris was pelleted by centrifugation at 10 000 *g* for 30 min at 4°C, and the supernatant was collected for immunoprecipitation with anti-CP190- and IgG-conjugated Protein G Magnetic beads (NEB, USA) (by incubating in the PBST on a rotary shaker at RT for 1 h) equilibrated in incubation buffer-150 (20 mM Tris–HCl, pH 7.4, with 150 mM NaCl, 10 mM MgCl_2_, 1 mM EDTA, 1 mM EGTA, 10% glycerol and 0.1% NP-40). The protein extract (50 μg protein) was adjusted to a volume of 500 μl with buffer-150, mixed with antibody-conjugated beads (30 μl), and incubated on a rotary shaker overnight at 4°C. The beads were then washed with two portions of buffer-150, two portions of incubation buffer-300 (with 300 mM NaCl), and one portion of incubation buffer-150, resuspended in SDS-PAGE loading buffer, boiled and analyzed by western blotting. Proteins were detected using the ECL Plus Western Blotting substrate (Thermo Fisher Scientific, USA).

### RT-PCR

Total RNA was isolated using the TRI reagent (Molecular Research Center, USA) according to the manufacturer's instructions. RNA was treated with two units of Turbo DNase I (Thermo Fisher Scientific, USA) for 30 min at 37°C to eliminate genomic DNA. The synthesis of cDNA was performed according to the manufacturer's instructions using 1 μg of RNA, 100 U of EpiScript reverse transcriptase (LGC Biosearch Technologies, UK) and 50 pM of oligo(dT) as a primer. The amounts of specific cDNA fragments were quantified by real-time PCR. At least three independent measurements were made for each RNA sample. Relative levels of mRNA expression were calculated in the linear amplification range by calibration to a standard genomic DNA curve to account for differences in primer efficiencies. Individual expression values were normalized with reference to *RpL32* mRNA. The primer used are listed in Supplementary Table S1.

### Cuticle preparation

Adult flies were collected in Eppendorf tubes and stored in 70% ethanol for at least 1 day. The ethanol was then replaced with 10% KOH, and the flies were heated at 70°C for 1 h. After heating, the flies were washed twice with dH_2_O and heated again in dH_2_O for 45 min. The digested flies were then washed with 70% ethanol and stored in 70% ethanol. The abdomen cuticles were cut from the rest of the digested fly using fine tweezers and an insulin syringe needle and placed in a drop of glycerol on a glass slide. The abdomens were then cut longitudinally on the dorsal side through all the tergites with the syringe. Some cuts were made between the tergites to ensure that the cuticle lay flat on the slide. The cuticles were then flattened with a coverslip. Photographs in the bright or dark field were taken on a Nikon SMZ18 stereomicroscope using Nikon DS-Ri2 digital camera, processed with ImageJ 1.50c4 and Fiji bundle 2.0.0-rc-46.

### Antibodies

Antibodies against the amino acids 44–200 of the Mzfp1 were produced in rabbits and rats, and purified by affinity purification on Aminolink resin (Thermo Fisher Scientific, USA) according to standard protocols. The other antibodies used were as follows: affinity purified rat anti-CP190 (33); affinity purified rabbit anti-Pita (33); affinity purified rabbit anti-Su(Hw) (84); mouse monoclonal anti-HA antibodies, clone HA-7 (#H3663, Sigma, USA); mouse monoclonal anti-lamin Dm0, clone ADL84.12 (#ADL84.12, DSHB, USA); mouse monoclonal anti-abd-A, clone C11 (sc-390990, Santa Cruz Biotechnology, USA).

### Immunostaining of polytene chromosomes

*Drosophila* 3rd instar larvae were cultured at 18°C under standard conditions. Polytene chromosome staining was performed as previously described (85). The following primary antibodies were used: rat anti-Mzfp1 at 1:100 dilution, rabbit anti-Mzfp1 at 1:100 dilution, mouse anti-HA at 1:100 dilution. The secondary antibodies were Alexa Fluor 488 goat anti-rabbit at 1:2000 dilution and Alexa Fluor 555 goat anti-mouse at 1:2000 dilution (Thermo Fisher Scientific, USA). The polytene chromosomes were co-stained with DAPI (AppliChem, Germany). Images were acquired on the Nikon Eclipse T*i* fluorescent microscope using Nikon DS-Qi2 digital camera, processed with ImageJ 1.50c4 and Fiji bundle 2.0.0-rc-46. Three to four independent stainings and 4–5 samples of polytene chromosomes were performed per transgenic line.

### Embryo immunostaining

Primary antibodies were mouse monoclonal anti-abd-A at 1:50 dilution. Secondary antibodies were goat anti-mouse Alexa Fluor 647 (Thermo Fisher Scientific, USA) at 1:2000 dilution. Stained embryos were mounted in the following solution: 23% glycerol, 10% Mowiol 4–88, 0.1M Tris–HCl pH 8.3. Images were acquired on Leica Stellaris 5 confocal microscope and processed using GIMP 2.8.16, ImageJ 1.50c4, Fiji bundle 2.0.0-rc-46.

### Protein extraction

Twenty adult flies were cooled in liquid nitrogen, homogenized for 30 sec with a pestle in 200 μl of extraction buffer (20 mM HEPES–KOH pH 7.5, 100 mM KCl, 5% glycerol, 10 mM EDTA, 1% NP-40, 1% sodium deoxycholate, 0.1% SDS, 1 mM DTT, 5 mM PMSF and 1:100 Calbiochem Complete Protease Inhibitor Cocktails VII and V) and incubated on ice for 10 min. The suspension was sonicated in a Bioruptor (Diagenode, USA) for 3 min on setting H, 15 s ON/45 s OFF. Then 4 × SDS-PAGE sample buffer was added to the homogenate. Extracts were incubated for 10 min at 100°C, centrifuged at 16 000 *g* for 5 min, and loaded on a 6 or 8% SDS-PAGE gel.

### Cellular fractionation

Sixty adult flies were homogenized with Potter and Dounce homogenizers (Wheaton) in 500 μl of ice-cold cytoplasm extraction buffer (CEB (50 mM HEPES–KOH pH 7.5, 10 mM NaCl, 1 mM EDTA, 10% glycerol, 0.5% NP-40, 0.25% triton X-100, 1 mM DTT and 1:100 Calbiochem Complete Protease Inhibitor Cocktails VII and V), filtered through a 70 μm cell strainer (Miltenyi Biotec, USA) and incubated on ice for 10 min. The homogenate was centrifuged at 3000 *g* at 4°C for 5 min.

The supernatant was collected in a fresh tube (cytoplasmic fraction), and the nuclear fraction was thrice washed with the same buffer. Then, the pellet was resuspended in 100 μl of ice-cold nuclear extraction buffer (NEB (10 mM Tris–HCl pH 8.0, 300 mM NaCl, 1 mM EDTA, 0.5 mM EGTA pH8.0 and 1:100 Calbiochem Complete Protease Inhibitor Cocktails VII and V), incubated on ice for 10 min and centrifuged at 3000 *g* at 4°C for 5 min. The supernatant was collected in a fresh tube (nucleoplasmic fraction), and the chromatin pellet was thrice washed with the same buffer. Then, the pellet was resuspended in 100 μl of ice-cold chromatin extraction buffer (ChEB (500 mM Tris–HCl pH 8.0, 500 mM NaCl and 1:100 Calbiochem Complete Protease Inhibitor Cocktails VII and V) and incubated on ice for 10 min. The solution (chromatin fraction) was sonicated in a Bioruptor (Diagenode, USA) for 5 min on setting H, 30 s ON/30 s OFF and centrifuged at 16 000 *g* at 4°C for 10 min. The supernatant was collected in a fresh tube (chromatin fraction). Then 4 × SDS–PAGE sample buffer was added to all the samples, and the extracts were boiled for 5 min at 100°C, centrifuged for 5 min at 16 000 *g*, and loaded on a 4–15%, 6% or 8% SDS–PAGE gel.

Protein samples were analyzed by immunoblot analysis. Proteins were detected using the SuperSignal West Femto Maximum Sensitivity Substrate (for detection of Mzfp1 in *y^1^w^1118^* line) or ECL Plus Western Blotting Substrate (Thermo Fisher Scientific, USA).

### Chromatin immunoprecipitation

Chromatin was prepared from 8 to 16 h embryos and 2- to 3-day-old adult flies. One gram of embryos was collected and fixed with formaldehyde as previously described (86). 500 milligrams of adult flies was ground in a mortar in liquid nitrogen and resuspended in 20 ml of buffer A (15 mM HEPES–KOH pH 7.6, 60 mM KCl, 15 mM NaCl, 13 mM EDTA, 0.1 mM EGTA, 0.15 mM spermine, 0.5 mM spermidine, 0.5% NP-40 and 0.5 mM DTT) supplemented with 0.5 mM PMSF and 1:500 Calbiochem Complete Protease Inhibitor Cocktail V. The suspension was then homogenized in a Potter and Dounce homogenizer with a tight pestle, filtered through a 100 μm Nylon Cell Strainer (Miltenyi Biotec, USA), and cross-linked with 1% formaldehyde for 15 min at room temperature. Cross-linking was stopped by adding glycine to a final concentration of 125 mM. The nuclei were washed with three 10-mL portions of wash buffer (15 mM HEPES–KOH pH 7.6, 60 mM KCl, 15 mM NaCl, 1 mM EDTA, 0.1 mM EGTA, 0.1% NP-40 and protease inhibitors) and one 5-ml portion of nuclear lysis basic buffer (15 mM HEPES pH 7.6, 140 mM NaCl, 1 mM EDTA, 0.1 mM EGTA, 1% Triton X-100, 0.5 mM DTT, 0.1% sodium deoxycholate, and protease inhibitors) and resuspended in 1 ml of nuclear lysis buffer (15 mM HEPES pH 7.6, 140 mM NaCl, 1 mM EDTA, 0.1 mM EGTA, 1% Triton X-100, 0.5 mM DTT, 0.1% sodium deoxycholate, 0.5% SLS, 0.1% SDS and protease inhibitors).

The suspension of embryo or adult flies nuclei was sonicated in a Covaris ME220 focused-ultrasonicator (40 alternating 15-s ON and 45-s OFF intervals, peak power 75, duty % factor 25), and 50-μl aliquots were used to test the extent of sonication and measure the DNA concentration. Debris was removed by centrifugation at 14 000 *g*, 4°C, for 10 min, and chromatin was pre-cleared with Protein A Dynabeads (Thermo Fisher Scientific, USA). Corresponding antibodies were incubated for 1 hour at room temperature with 20 μl aliquots of Protein A (rabbit anti-Mzfp1, 1:100; anti-Pita, 1:500; anti-Su(Hw), 1:300; rabbit non-specific IgG) or G (rat anti-Mzfp1, 1:100; anti-CP190, 1:300; rat non-specific IgG) Dynabeads (Thermo Fisher Scientific, USA) mixed with 200 μl of PBST. Then antibody–Dynabeads complexes and anti-HA Magnetic beads (Thermo Fisher Scientific, USA) were washed and equilibrated in nuclear lysis buffer. Chromatin samples containing 10–20 μg of DNA equivalent in 200 μl nuclear lysis buffer (2 μl aliquots of pre-cleared chromatin as input material) were incubated overnight at 4°C with antibody–Dynabeads complexes. After three rounds of washing with lysis buffer supplemented with 500 mM NaCl and TE buffer (10 mM Tris–HCl pH 8.0; 1 mM EDTA), the DNA was eluted with elution buffer (50 mM Tris–HCl pH 8.0, 1 mM EDTA, 1% SDS), the cross-links were reversed, and the precipitated DNA was extracted using a ChIP DNA Clean & Concentrator kit (Zymo Research, USA).

The enrichment of specific DNA fragments was analyzed by qPCR, using a QuantStudio 6 Thermal Cycler (Thermo Fisher Scientific, USA). The primer used are listed in Supplementary Table S1. To normalize the results obtained from the different fly lines, genomic regions were used for which peaks with binding sites of the corresponding proteins were reproducibly detected, and the level of enrichment in values was comparable to the native element *Fub*: for Pita - 50E, for Su(Hw) - 62D, for Mzfp1 and CP190 - 94C. The *rpl32* region was used as a negative control of protein binding.

The ChIP-seq libraries were prepared with the NEBNext®_Ultra™_II DNA Library Prep kit per the manufacturer's instructions. Amplified libraries were quantified using fluorometry with DS-11 (DeNovix, USA) and a Bioanalyzer 2100 (Agilent, USA). Diluted libraries were clustered on a pair-read flowcell and sequenced using a NovaSeq 6000 system (Illumina, USA).

### ChIP-seq data processing and sequence analysis

Chromatin immunoprecipitation sequencing (ChIP-seq) analysis was performed for 14 samples:

- Mzfp1 in *y^1^w^1118^* embryos with anti-Mzfp1 antibodies raised in rat (single-end);
- Mzfp1 in *y^1^w^1118^* embryos with anti-Mzfp1antibodies raised in rabbit (single-end);
- Preimmune control in embryos with non-specific antibodies raised in rat (single-end);
- Preimmune control in embryos with non-specific antibodies raised in rabbit (single-end);
- Mzfp1 in adult flies with anti-HA antibodies (Mzfp1^wt^-HA anti-HA, paired-end);
- Mzfp1 in adult *y^1^w^1118^* flies with anti-Mzfp1 antibodies raised in rat (paired-end);
- Preimmune control in adult flies with anti-HA antibodies (paired-end);
- Mzfp1 in adult flies with anti-Mzfp1 antibodies raised in rat (Mzfp1^wt^-HA anti-Mzfp1, paired-end);
- Preimmune control in adult flies with non-specific antibodies raised in rat (paired-end);
- Mzfp1^ΔM2^-HA in adult flies with anti-HA antibodies (Mzfp1^ΔM2^-HA anti-HA, paired-end);
- Mzfp1^ΔZF^-HA in adult flies with anti-HA antibodies (Mzfp1^ΔZf^-HA anti-HA, paired-end);
- Mzfp1^Δ42^-HA in adult flies with anti-HA antibodies (Mzfp1^Δ42^-HA anti-HA, paired-end);
- Mzfp1^ΔM2^-HA in adult flies with anti-Mzfp1 antibodies raised in rat (Mzfp1^ΔM2^-HA anti-Mzfp1, paired-end);
- Mzfp1^Δ42^-HA in adult flies with anti-Mzfp1 antibodies raised in rat (Mzfp1^Δ42^-HA anti-Mzfp1, paired-end).

The data are available in the NCBI Gene Expression Omnibus (GEO) under accession number GSE242677. Two biological replicates were obtained for each sample (except preimmune controls and Mzfp1 in embryos with antibodies raised in rat and rabbit (the last two were considered as replicates themselves)). Reads were processed as described previously (87), the main steps were:

- trimming Illumina adapters with cutadapt (parameters: -a “T{100}”, -m 20 –trim-n –minimum-length=20; for paired-end samples: –pair-filter=any) (88);
- trimming low quality ends with sickle (parameters: -q 20 -l 20 -n) (https://github.com/najoshi/sickle);
- alignment with bowtie2 (parameters: –no-discordant –no-mixed) (89) for paired-end samples and with bowtie (90) (parameters: –best –strata -m 1 –tryhard) for single-end samples aganst version dm6 of the *Drosophila melanogaster* genome;
- filtration of PCR duplicates and non-unique mapping with picard (picard functions FilterSamReads and MarkDuplicates) (https://broadinstitute.github.io/picard/);
- blacklist filtration with bedtools (91);
- reproducible peaks calling against corresponding preimmune controls with IDR pipeline (https://sites.google.com/site/anshulkundaje/projects/idr) for all sampes exept two (Mzfp1 in embryos with antibodies raised in rat and rabbit). A soft pvalue threshold for MACS2 in IDR of 0.01 was used (other MACS2 parameters: –gsize=dm –tsize=101 –format BAMPE). The IDR threshold was set to 0.05 for true replicates and 0.01 for pseudoreplicates;
- Peak calling Mzfp1 in embryos with antibodies raised in rat and rabbit against corresponding preimmune controls with MACS2 (parameters: –gsize=dm –tsize=51 –pvalue=1×10-5) (92).

Mzfp1^ΔM2^-HA anti-HA, Mzfp1^ΔZF^-HA anti-HA, Mzfp1^Δ42^-HA anti-Mzfp1 and *y^1^w^1118^* anti-Mzfp1 adult showed good reproducibility (both the rescue ratio and the self consistency ratio were <2) while Mzfp1^Δ42^-HA anti-HA, Mzfp1^wt^-HA anti-HA, Mzfp1^ΔM2^-HA anti-Mzfp1 and Mzfp1^wt^-HA anti-Mzfp1 showed moderate reproducibility (one of rescue ratio or self consistency ratio were more than 2) (Supplementary Figure S1). Therefore all samples with two biological replicates passed the reproducibility analysis. Using samples with one biological replicate (Mzfp1 in embryos with antibodies raised in rat and rabbit) we obtained a union set of Mzfp1 peaks in embryos using findOverlapsOfPeaks function from the ChIPpeakAnno R package (93).

ChiP-seq coverage tracks (BedGraph) were obtained using deepTools (94) bamCoverage function, with a bin width of 50 bp and RPKM normalization. Genomic tracks were visualized using svist4get software (95). *De novo* motif discovery was performed using ChIPMunk (96,97). For motif discovery the top-100, top-200 and top-500 peaks per sample were narrowed to ±200 bp around the summit, ChIPMunk was run in peak summit mode, motif length was set to 15. Motif was discovered for union set of Mzfp1 peaks in embryos. Genome-wide motif sites were identified using sarus (https://github.com/VorontsovIE/sarus) with *P* value threshold 1 × 10^−4^. We considered that a binding site contains a motif if *P* value was less than 1 × 10^−4^ for at least one of the top-100 or the top-500 motif.

Downstream analysis was performed in R statistical programming language, version 4.2.2 (98). Peak annotation was performed using ChIPseeker (99) and GenomicRanges (100) packages; promoter segments were considered as ±200 bp from the TSS. Analysis of the overlapping peaks was performed with ChIPpeakAnno (93) and visualized using UpSetR package (101).

Heterochromatic regions were identified using centromeres coordinates for build genome version dm6 (2L:22000975-23513712; 2R:1:5398184, 3L:22962476-28110227, 3R:1-4174178, X:22628490-23542271) (102). Housekeeping genes were obtained from the previously published data (103).

Colocalization of Mzfp1 binding sites with other proteins (M1BP, Su(Hw), ZIPIC, Pita, dCTCF, BEAF-32 and CP190) was made using publicly available ChIP-Seq data. For each of the proteins we obtained raw reads and corresponding controls and processed them as described above. The following data were used: M1BP (39), Su(Hw) (104), ZIPIC (32), Pita (32), dCTCF (87), BEAF-32 (105) and CP190 (64). For all proteins (except CP190) only peaks with a corresponding motif site (sarus *P* value < 1×10^−4^) were used in the analysis.

We also investigated the colocalization between binding sites of the pairs of proteins listed above in the promoters of housekeeping genes. To determine whether the observed fraction of shared sites (Fo) is higher than expected by chance, we used the Monte Carlo method. For each protein pair (P1 with N1 binding sites in housekeeping gene promoters and P2 with N2 such sites), we randomly sampled 5000 pairs of random sets (with sizes N1 and N2) of housekeeping promoters without replacement. For each of these 5000 random sets pairs, we calculated the ratio between shared promoter IDs and all IDs included in the sets. These simulations provided us with the distribution of the fraction of overlapping sites (Fs) for a specific protein pair expected by chance. Subsequently, we computed the p-value as the proportion of Fs values that exceeded the observed Fo value. After obtaining *P* values for each pair of proteins, we conducted multiple comparison correction with the Benjamini–Hochberg method.

Gene ontology enrichment analysis (overrepresentation analysis) was performed using the enrichGO function from clusterProfiler package (106) with biological processes (BP) subontology and Benjamini–Hochberg correction for multiple comparison.

### RNA-seq

Total RNA was isolated using the TRI reagent (Molecular Research Center, USA) according to the manufacturer's instructions. mRNA was isolated using oligo(dT) Magnetic beads (NEB, USA) according to the manufacturer's instructions. RNA-Seq libraries were prepared with the NEBNext® Ultra RNA Library Prep kit (NEB, USA) per the manufacturer's instructions. Amplified libraries were quantified using fluorometry with DS-11 (DeNovix, USA) and a Bioanalyzer 2100 (Agilent, USA). Diluted libraries were clustered on a pair-read flowcell and sequenced using a NovaSeq 6000 system (Illumina, USA).

RNA-Seq was performed for two samples: *y^1^w^1118^* (two biological replicates) and *CG1603^attP^* (three biological replicates) 3rd instar larvae. The data are available in the NCBI Gene Expression Omnibus (GEO) under accession number GSE242679. Adapters, poly-A tails and low-quality ends were processed as described above for ChIP-Seq data. The processed reads were aligned to the version dm6 of the *Drosophila melanogaster* genome using the splice-aware STAR algorithm (107). The gene read counts were calculated using the STAR quantMode option (–quantMode GeneCounts).

Further analysis was performed in R version 4.2.2 (98). Firstly, the read counts were normalized using the TMM algorithm (108) after discarding the reads with multiple mapping (multimapping), those overlapping with more than one gene (ambiguous), not overlapping with genes (noFeature), unmapped read (unmapped) and mapped on ribosomal RNA. Pearson correlation between biological replicates after normalization was >0.97 (Supplementary Figure S2). Then, the differentially expressed genes were identified using the estimateDisp, glmQLFit and glmQLFTest functions from the edgeR package (109) with 0.05 FDR significance threshold.

## Results

### Mzfp1 directly interacts with CP190

**Figure 1.**
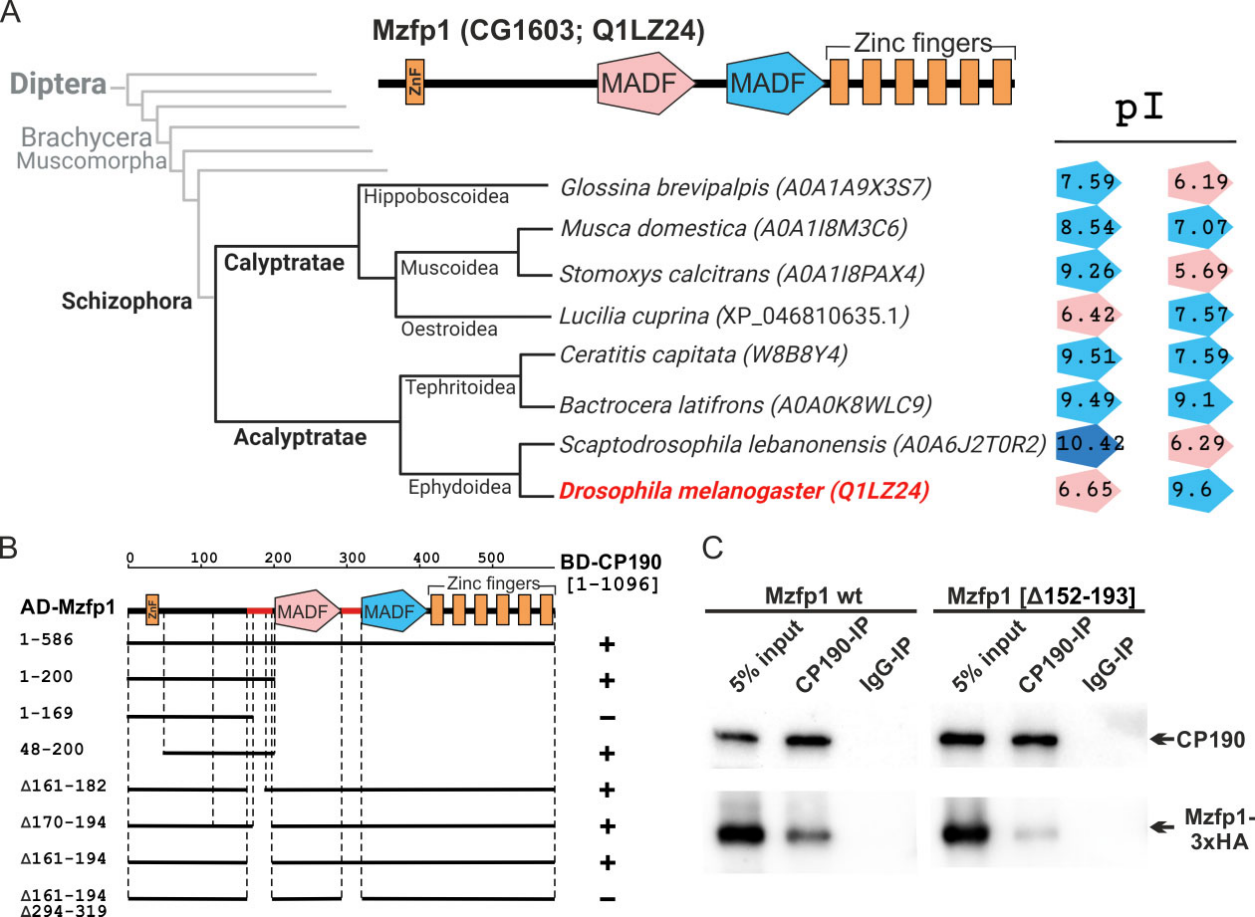
Mzfp1 is a new C2H2 protein that interacts with CP190. (**A**) Phylogenetic tree with species of the Schizophora section of Diptera that contain Mzfp1 homologous proteins. A schematic representing the general organization of the Mzfp1 protein of *Drosophila melanogaster* is shown at the top. Two MADF domains (pink and blue pentagons) and C2H2 domains (orange rectangles) are indicated. The right panel indicates MADF domains that are contained in annotated proteins with the associated isoelectric points. The colors of the pentagons identify the MADF domains by their charges: blue = positively charged, pink = negatively charged. (**B**) Mapping of Mzfp1 domains interacting with CP190 in a yeast two-hybrid assay. Different fragments of Mzfp1 were fused to the GAL4 activating domain and tested for interaction with CP190 fused to the GAL4 DNA-binding domain. All Mzfp1 fragments were tested for the absence of interaction with the GAL4 DNA-binding domain alone (BD). The results are summarized in the columns on the right, with the ‘+’ and ‘–‘ signs referring to the presence and absence of interaction, respectively. Potential regions in Mzfp1 that interact with CP190 are indicated by red lines. (**C**) Total extracts from *Drosophila* S2 cells co-transfected with CP190 and Mzfp1-3xHA expressing plasmids were immunoprecipitated with antibodies against CP190 or nonspecific IgG as a negative control, and the immunoprecipitates were analyzed by Western blotting for the presence of HA-tagged proteins. CP190 and IgG immunoprecipitates are shown concentrated relative to the input by a factor of 20. Created with BioRender.com.

The Mzfp1 (CG1603) protein was initially identified as interacting with Chromator (*Chriz*/*Chro* gene) and Z4 (*pzg/Z4* gene) proteins in a co-affinity purification screen assay coupled with mass spectrometry analysis (110). Chromator and Z4 in cooperation with CP190 are associated with housekeeping gene promoters (111–113). Therefore, we selected Mzfp1 as a candidate C2H2 protein that could be involved in the organization of housekeeping promoters, as previously demonstrated for the M1BP protein (38–40). The Mzfp1 protein (Figure 1A) is composed of 586 aa and belongs to a small group of C2H2 proteins having an unusual structure. The protein contains an N-terminal zinc-finger domain of the C2H2 type (18–43 aa), an unstructured linker, two MADF domains named MADF1 (201–292 aa) and MADF2 (320–413 aa), and a C-terminal cluster consisting of six C2H2 domains (418–583 aa) (Figure 1A). The negatively charged MADF1 (isoelectric point 6.65) can be involved in recruiting transcriptional complexes, while the positively charged MADF2 (isoelectric point 9.6) can bind to DNA, as in many other characterized positively charged MADF domains (114). The Mzfp1 protein is conserved among species of Drosophilidae that usually have an orthologous protein with the (C2H2)-MADF1-MADF2-(C2H2)_6_ organization (Supplementary Figure S3). MADF domains of the orthologous proteins from the closest species of *Drosophila* (predominantly from the *melanogaster* group) have the same physico-chemical properties (p*I* for MADF1 = 6.2–6.8; for MADF2 = 8.5–9.6). Orthologous proteins from more distant *Drosophila* species are characterized by the retention of the two MADF domains in the proteins, but the charges in different groups vary significantly (Supplementary Figure S4A). For example, in many cases both MADF domains are positively charged (the *willistoni*, *repleta* and *virilis* groups). At the same time, the *obscura* group is characterized by a strongly acidic MADF1 domain. A search among other Dipteran families also revealed several orthologous proteins in the closest relatives in the Schizophora section (Figure 1A and Supplementary Figure S5). Despite the extremely low level of homology, the general organization of homologous proteins was preserved in all cases ((C2H2)-MADF1-MADF2-(C2H2)_6-7_) (Supplementary Figure S5), while the MADF domains are characterized by very different isoelectric points (Figure 1A). Finally, four more paralogous proteins with an organization similar to Mzfp1 were found in the *Drosophila melanogaster* genome (Supplementary Figure S4B). All such proteins, with the exception of CG8944, contained both types of MADF domains, i.e. acidic and basic.

To examine the direct interactions of Mzfp1 with CP190, Z4, and Chrom, we used a yeast two-hybrid (Y2H) assay. The results demonstrated a direct interaction only between Mzfp1 and CP190 (data not shown). Next, we determined the domains involved in the interaction between CP190 and Mzfp1. Different Mzfp1 fragments were cloned with a GAL4 activation domain (AD) and then tested against CP190 fused with a GAL4 DNA-binding domain (DBD). Based on the results, we mapped two potential CP190-interacting regions in the Mzfp1 protein that include 161–194 aa and 294–319 aa (between the MADF domains) (Figure 1B, Supplementary Figure S6A). We also tested the interacting domains in CP190 (1096 amino acids) that contain an N-terminal BTB/POZ domain (30–127 aa), two centrosomal targeting domains, four C2H2 zinc-finger domains (470–590 aa), and a C-terminal D/E-rich domain (592–1096 aa) (Supplementary Figure S6B) (115–117). Well-known insulator proteins like Su(Hw), dCTCF and Pita, primarily interact with the BTB domain (43), while three previously characterized M1BP, Opbp, and ZIPIC proteins, that bind preferentially to the promoters of housekeeping genes, interact with two CP190 regions, 210–245 aa and 309–390 aa (118). The different fragments of the CP190 protein were tested with Mzfp1, Mzfp1(Δ161–194), and Mzfp1(1–200) (Supplementary Figure S6B,C). As a result, we found that both regions of Mzfp1 interact with the 210–245 aa region of CP190. In addition, Mzfp1(1-200) interacts with the 309–390 aa region of CP190.

Finally, we confirmed the interaction between the proteins by coimmunoprecipitation of CP190 and 3×HA-tagged Mzfp1 transfected into S2 cells (Figure 1C and Supplementary Figure S7). Interestingly, 3×HA-tagged Mzfp1(Δ152-194) still weakly interacts with CP190 in coimmunoprecipitation confirming that the 294–319 aa region of Mzfp1 is also involved in interaction with CP190.

### Mzfp1 primarily binds to housekeeping gene promoters located in both euchromatin and heterochromatin

To further characterize Mzfp1, we generated polyclonal affinity-purified antibodies in both rats and rabbits against a 157 amino acid protein region (44–200 aa) (Supplementary Figure S8A). These antibodies were used to identify overlapping banding patterns on polytene chromosomes from larval salivary glands (Supplementary Figure S8B). The rabbit and rat antibodies predominantly stained pericentromeric heterochromatin and a number of interbands in the euchromatin, suggesting that Mzfp1 binds to both euchromatin and heterochromatin regions. Notably, the rat antibodies stained the euchromatin regions of polytene chromosomes more intensely than the rabbit antibodies.

**Figure 2.**
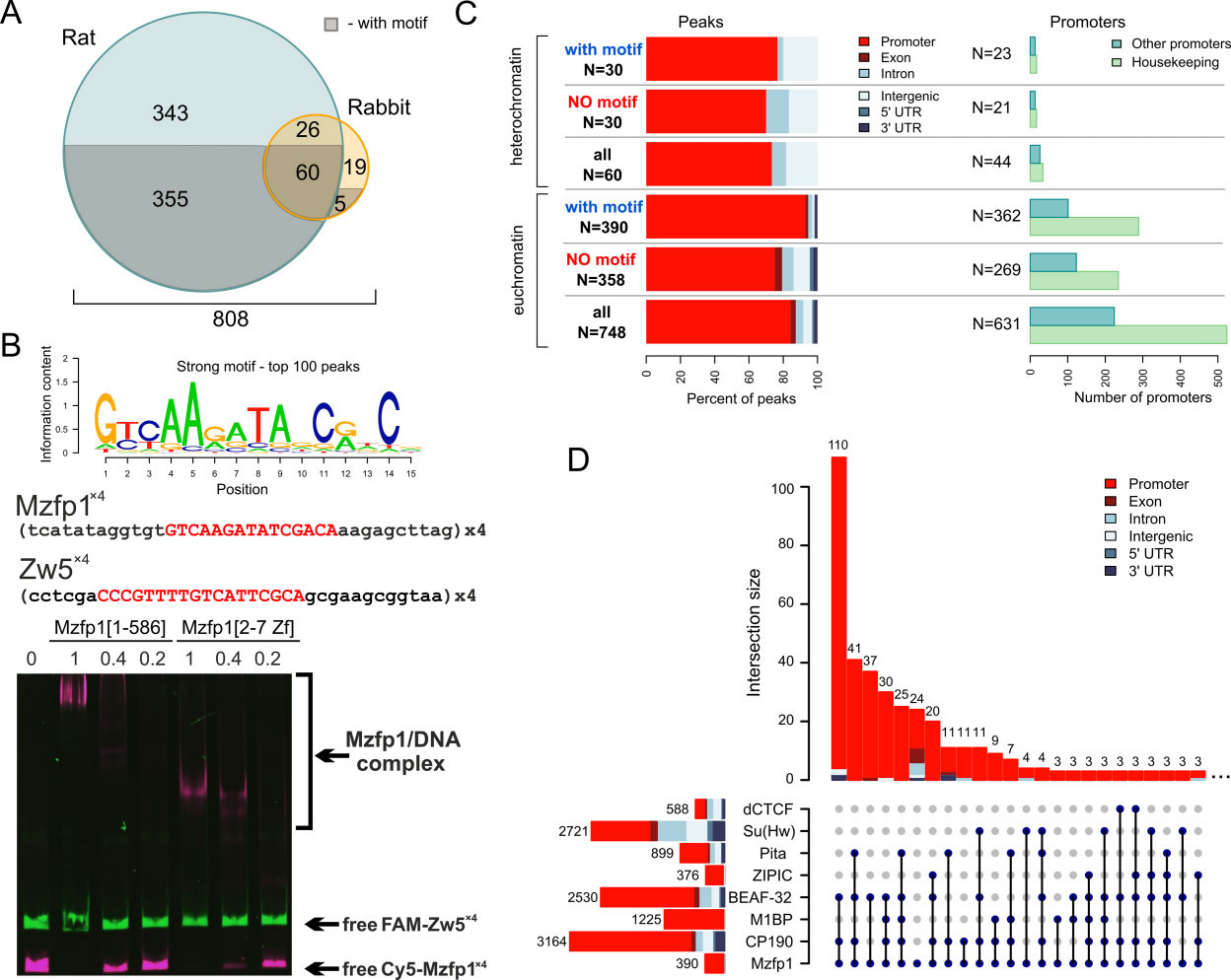
Mzfp1 binds to promoters of housekeeping genes. (**A**) Venn diagram for Mzfp1 binding sites (with and without the motif) obtained with antibodies against Mzfp1 obtained in rats and rabbits. (**B**) The consensus binding site (Mzfp1 motif) identified by ChIPMunk for the top 100 peaks from the combined set of Mzfp1 binding sites obtained using rat and rabbit antibodies. Below is the electrophoretic mobility shift assay for the recombinant full-sized Mzfp1 as well as for the cluster of C2H2 domains from Mzfp1 only. MBP-fused Mzfp1 was incubated with fluorescently labeled DNA fragments: four Mzfp1 binding sites (Mzfp1^x4^) labeled with Cy5 and four Zw5 binding sites (Zw5^x4^) labeled with fluorescein amidite (FAM) (used as a negative control). Signals were detected for FAM-labeled fragments at an excitation wavelength of 500 nm and an emission wavelength of 535 nm and for the Cy5-labeled fragment at an excitation wavelength of 630 nm and an emission wavelength of 700 nm. (**C**) The distribution of Mzfp1 binding sites (with and without a motif) by genomic elements in euchromatic and heterochromatic regions of the genome is shown on the left. On the right, the distribution of Mzfp1 sites between promoters of housekeeping genes and other *Drosophila* promoters is shown. (**D**) Colocalization of Mzfp1 sites with the binding sites for the M1BP, dCTCF, Su(Hw), Pita, ZIPIC, BEAF-32 and CP190 proteins. Only binding regions with motifs for all proteins except CP190 were considered in this analysis. The bars and numbers on the left correspond to all binding sites included in the analysis for the each protein.

To determine the binding sites of Mzfp1 *in vivo*, we performed chromatin immunoprecipitation followed by next-generation sequencing (ChIP-seq) on chromatin extracted from 0 to 12 h embryos. We conducted two independent biological replicates using rat and rabbit antibodies. We identified 784 high-confidence peaks with rat antibodies and 110 peaks with rabbit antibodies (Figure 2A), with 86 peaks common to both data sets. *De novo* motif discovery using ChIPMunk identified a new sequence motif, a 15 bp element (Figure 2B and Supplementary Figure S9) that is common among transcription factors with C2H2 domain clusters. The specific binding of Mzfp1 to four copies of the motif (Mzfp1^x4^) was confirmed *in vitro* by EMSA using the bacterially expressed recombinant full-sized Mzfp1 protein or a cluster of C2H2 domains only (Figure 2B).

The distribution of Mzfp1 peaks was correlated with genomic annotations, and we found that the majority (more than 70%) of the protein binding sites were located in promoter regions of both euchromatic and heterochromatic genes (Figure 2C, Supplementary Table S2). Moreover, almost all euchromatic peaks with motifs (>95%) were located in the promoters. The binding motifs were located in the ±200-bp region near the transcriptional start site (TSS), suggesting the involvement of the protein in promoter organization. More than 60% of the Mzfp1-dependent euchromatic promoters regulated housekeeping genes (Supplementary Table S3). Only a small number of Mzfp1 sites were located in intergenic regions (<2% of euchromatic sites with a motif).

To study the colocalization of Mzfp1 with CP190 and well-characterized architectural C2H2 proteins in euchromatic regions (Figure 2D), we included previous data for M1BP (39), Su(Hw) (104), Pita, ZIPIC (32), dCTCF (87), BEAF-32 (119,120), and CP190 (64). All tested architectural C2H2 proteins have been shown to interact with CP190 (33,40,42,46,87,112,121). In this study, we only tested motif-containing binding sites for the C2H2 architectural proteins for colocalization with Mzfp1. Our analysis (Figure 2D and Supplementary Figure S10) revealed an unusually high level of colocalization between Mzfp1, CP190 (78% of sites) and BEAF-32 (83% of sites) in the promoter regions, indicating close cooperation between them. Since M1BP is known to be the main architectural protein that binds to housekeeping gene promoters (38,40), we expected significant colocalization of Mzfp1 (21%) with M1BP and CP190. Surprisingly, we also found strong colocalization between Mzfp1/CP190 and Pita (22% of the total number of Mzfp1 sites). Furthermore, 32 CP190-dependent promoters contained binding sites for Mzfp1, Pita, and M1BP simultaneously. Additionally, we observed colocalization between Mzfp1 and ZIPIC in 34 promoters (Figure 2D). The high level of colocalization between the binding sites of these tested C2H2 proteins supported the hypothesis that each promoter is associated with several architectural C2H2 proteins that recruit CP190.

### Mzfp1 is essential for *Drosophila* viability

**Figure 3.**
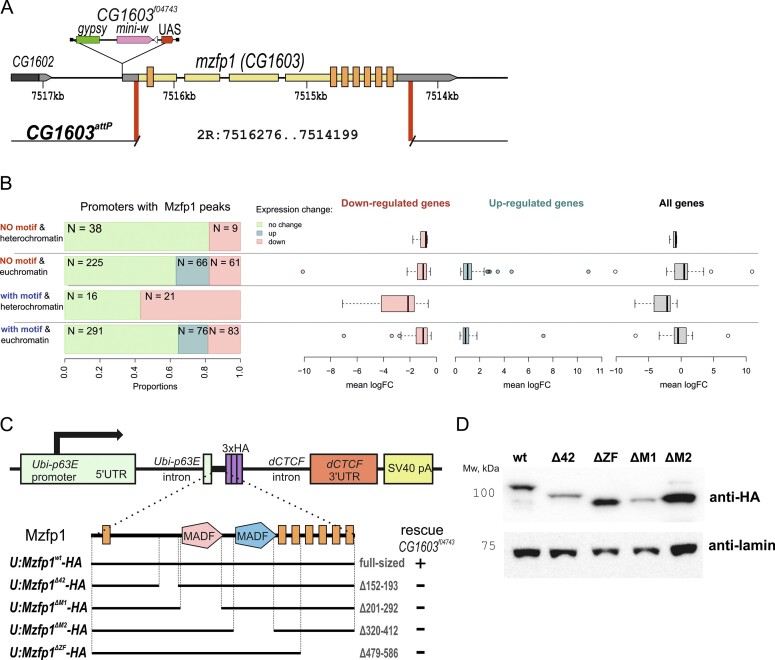
The role of *Mzfp1 in vivo*. (**A**) A schematic representation of the *CG1603^f04743^* mutation and the *CG1603^attP^* deletion. (**B**) Expression comparison using RNA-seq analysis in *wild-type* and *CG1603^attP^*/*CG1603^attP^*larvae of euchromatic and heterochromatic groups of genes containing the Mzfp1 binding site with (‘with motif’) or without (‘NO motif’) the motif in the promoter region. The *y^1^w^1118^; CG1603^attP^*/*CG1603^attP^* larvae were collected from the cross between *y^1^w^1118^; CG1603^attP^/СyO GFP* males and females. The proportions of differentially expressed genes among those containing Mzfp1 binding sites in the promotor region (on the left) and the mean log-fold change for down-regulated, up-regulated and all differentially expressed genes containing Mzfp1 binding sites in the promotor region are shown on the right. (**C**) Schematic representation of the deletions in the Mzfp1 protein. A schematic showing the construct used to express wild-type Mzfp1 under the control of the *Ubi-p63E* (*U*) promoter in a transgenic *Drosophila* line. Green boxes – promoter and 5’UTR of *Ubi-p63E* gene; red box – 3’UTR with polyadenylation signal from *dCTCF* gene; yellow box – polyadenylation signal from virus SV40; violet boxes – 3xHA epitope. The black lines correspond to introns. The transgenes expressing different variants of Mzfp1 were tested in heterozygous state in combination with the *CG1603^f04743^* homozygote. (**D**) Immunoblot analysis (8% SDS-PAGE) of protein extracts from transgenic flies expressing wild-type and deletion variants of Mzfp1 in adult flies (wt (Mzfp1^wt^-HA), Δ42 (Mzfp1^Δ42^-HA), ΔZF (Mzfp1 ^ΔZF^-HA), ΔM1 (Mzfp ^ΔM1^-HA), ΔM2 (Mzfp ^ΔM2^-HA)) with antibodies against HA-epitope. Antibodies for lamin Dm0 were used as an internal control.

To determine the functional role of Mzfp1, we used a previously characterized mutation, *CG1603^f04743^* (BL18801), in the *mzfp1* (*CG1603*) gene that was obtained in the Gene Disruption Project (122). The *CG1603^f04743^* mutation was generated by the insertion of the *pBac* transposon in the *5’UTR* of *mzfp1* at 70 bp from the coding region (Figure 3A and Supplementary Figure S11). The *CG1603^f04743^* mutation was balanced with *CyO,GFP* (BL4533), because the *CG1603^f04743^*homozygotes died in the second-instar or less often in the third-instar larval stage. Next, we deleted the *CG1603* gene in the *y^1^w^1118^* line (Figure 3A and Supplementary Figure S11A) using CRISPR/Cas9 technology (78,123,124). As a result, the 2078 bp of the *CG1603* gene was substituted with the *attP* site and a *3P3:dsRed* reporter flanked by *loxP* sites, *CG1603^attP^* (Supplementary Figure S11A). The obtained deletions were balanced with *CyO,GFP*. The offspring obtained from crossing *CG1603^attP^* with *CG1603^f04743^*or homozygous *CG1603^attP^* died during the second or the third-instar larval stage. Thus, we conclude that Mzfp1 is essential for *Drosophila* development. We also used *CG1603^attP^/CG1603^attP^*larvae to confirm the specificity of anti-Mzfp1 antibodies (Supplementary Figure S12). To test distribution of Mzfp1 in cells, we compared the amount of Mzfp1 and CP190 in the cytoplasmic, nucleoplasmic, and chromatin fractions of *y^1^w^1118^* flies (Supplementary Figure S13). Both antibodies detected approximately equal amounts of Mzfp1 in the nucleoplasm and in association with chromatin. In contrast, the CP190 protein, which does not interact directly with DNA, was predominantly detected in the nucleoplasm fraction.

To test the functional role of Mzfp1 in the regulation of promoters, we compared the expression of a selected group of genes in *y^1^w^1118^* and *y^1^w^1118^; CG1603^attP^/CG1603^attP^* larvae by RNA-seq (Figure 3B, Supplementary Figure S2 and Supplementary Table S4). For the analysis, we chose genes that demonstrated a constant level of expression during development and that contained either Mzfp1 peaks with motifs or Mzfp1 peaks only in the promoter regions. We found that Mzfp1 inactivation affected the expression of 21 out of 37 genes (60%) in heterochromatin that had the Mzfp1 motif in their promoters. In contrast, Mzfp1 inactivation had a moderate effect on euchromatin promoter transcription, showing slight increases or decreases in approximately the same number of genes, about 20% (Figure 3B and Supplementary Table S4). Thus, Mzfp1 is essential for the activity of heterochromatin promoters, most of which control the expression of important housekeeping genes (Supplementary Table S4).

It seems likely that in euchromatic promoters, Mzfp1 binds in combination with other architectural C2H2 proteins and hence may have redundant functions.

### The CP190 interacting region and MADF domains are essential for the activity of Mzfp1 *in vivo*

To study the functional role of Mzfp1 domains, we created a transgenic line in which the cDNA of Mzfp1 fused with 3×HA (Mzfp1^wt^-HA) was expressed under the control of the strong *Ubiquitin-63E* (*U*) promoter (*U:Mzfp1^wt^*) (Figure 3C). The last of the C2H2 domains of Mzfp1 is located directly at the C-terminus, and therefore we fused the HAx3-tag with the C-terminal domain via a 23 aa linker. We inserted the construct into the 86F8 region of the *y^1^w^1118^* line using a *φC31*-integration system (81,125). We crossed the *U:Mzfp1^wt^* transgene into the *CG1603^f04743^*/*CG1603^attP^* background, and flies *U:Mzfp1^wt^*/*TM6,Tb*; *CG1603^f04743^*/*CG1603^attP^*displayed a wild-type (*wt*) phenotype, demonstrating that Mzfp1^wt^-HA was functional.

Mzfp1 has two potential DNA binding regions: the C-terminal cluster of C2H2 domains and the positively charged MADF2 domain (Figure 3C). To clarify the functional roles of individual domains, we produced deletions in the Mzfp1: CP190 interacting region (Δ42), MADF1 (ΔM1), MADF2 (ΔM2) and the C2H2 domains from 3 to 6 from the C-terminal cluster (ΔZF). We obtained transgenic lines at the 86F8 region in the same way as for the *U:Mzfp1^wt^* transgene (Figure 3C). Immunoblot analysis (Figure 3D and Supplementary Figure S14A) showed that Mzfp1^wt^-HA and Mzfp1^ΔZF^-HA variants were expressed at relatively similar levels in adult flies. The expression level of Mzfp1^ΔM2^-HA was higher, while expression of Mzfp1^ΔM1^-HA and Mzfp^Δ42^-HA was lower than that of Mzfp1^wt^-HA.

As transcription of all transgenes was at similar levels (Supplementary Figure S14B), the deletion of the CP190 interacting region and MADF1 domain or MADF2 domain likely affected the stability of the mutant Mzfp1 in opposite ways. At the same time, the expression level of Mzfp1^wt^-HA in the transgenic line was higher than that of wild-type Mzfp1 in *y^1^w^1118^* line (Supplementary Figure S8A). Moreover we could detect only very weak band in the total protein extract from *y^1^w^1118^* line with immunoblot analysis. For this reason, we compared the expression of Mzfp1 in the cytoplasmic, nucleoplasmic, and chromatin fractions obtained from the 2–3 day-old adults of the *y^1^w^1118^* and *U:Mzfp1^wt^*/*TM6,Tb*; *CG1603^attP^*/*CG1603^attP^*(*Mzfp1^wt^*) lines (Supplementary Figure S15). Only very faint bands corresponding to Mzfp1 were detected in the cytoplasmic fraction for *y^1^w^1118^* and *U:Mzfp1^wt^*/*TM6,Tb*, indicating an exclusively nuclear localization of the protein. In both lines, Mzfp1 is evenly distributed between nuclear and chromatin fractions. The amount of Mzfp1 in the *Mzfp1^wt^* line is several times greater than in the *y^1^w^1118^*.

**Figure 4.**
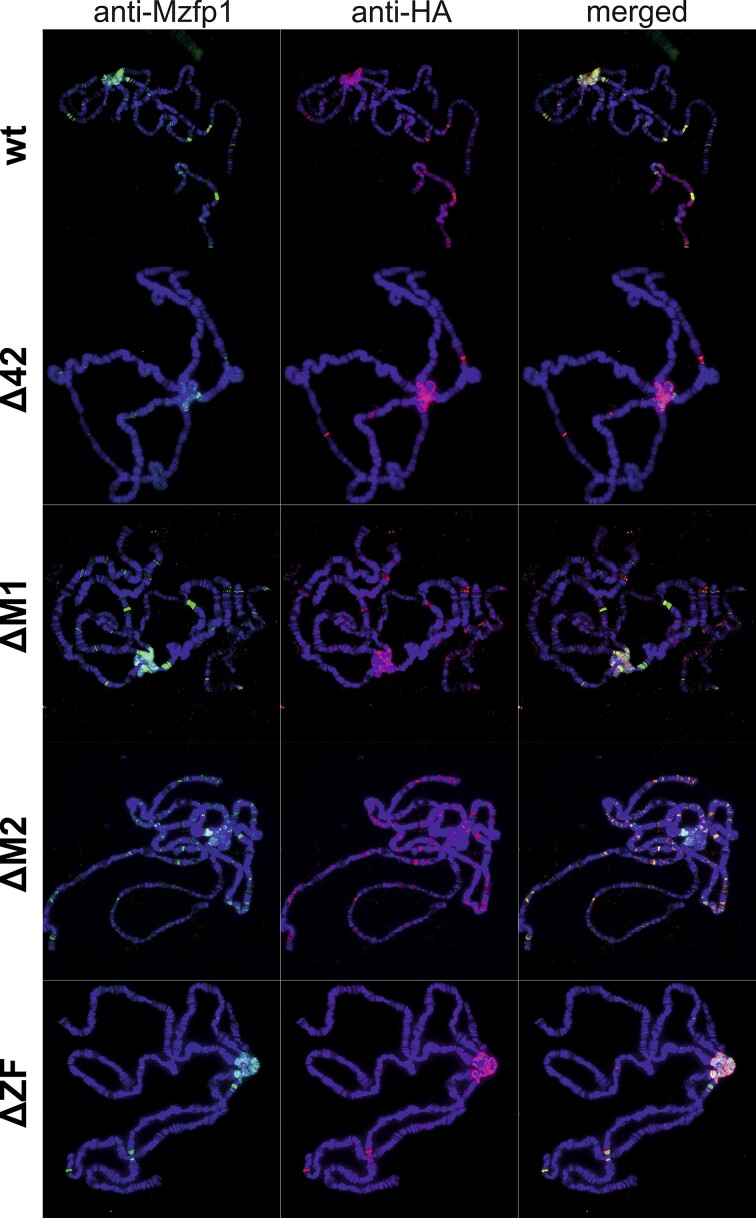
Distribution of the Mzfp1-HA variants (wt (Mzfp1^wt^-HA), Δ42 (Mzfp1^Δ42^-HA), ΔM1 (Mzfp ^ΔM1^-HA), ΔM2 (Mzfp ^ΔM2^-HA), and ΔZF (Mzfp1 ^ΔZF^-HA)) on polytene chromosomes from larval salivary glands. The panels show the merged results of immunostaining of endogenous Mzfp1 and Mzfp1-HA variants (green, rabbit anti-Mzfp1 antibody; red, mouse anti-HA antibody). DNA was stained with DAPI (blue).

All obtained transgenes expressing mutant variants of Mzfp1 failed to complement *CG1603^f04743^*/*CG1603^attP^* or *CG1603^attP^*/*CG1603^attP^*suggesting that the MADF domains, the CP190-interacting region, and the C2H2 cluster are essential for protein activity (Figure 3C). Next, we used antibodies against Mzfp1 and HA-epitope to compare the binding of Mzfp1-HA and its mutant derivatives to polytene chromosomes of third-instar larval salivary glands. In homozygotes for the *CG1603^−^* mutations expressing any of the Mzfp1-HA* variants, rare larvae surviving to 2–3 stages had the polytene chromosomes with poor morphology. For this reason, we examined polytene chromosomes in CG1603^attP^/CyO; U:Mzfp1*/ TM6,Tb lines (Figure 4).

Despite significantly stronger Mzfp1 expression in the CG1603^attP^/CyO; U:Mzfp1*/ TM6,Tb flies in comparing with *y^1^w^1118^* flies, patterns of Mzfp1 binding in euchromatin regions of polytene chromosomes was the same in larvae of both lines, suggesting that overexpression of Mzfp1 does not affect specificity of its binding to euchromatic sites. In addition to binding to a relatively small number of bands in euchromatin regions, the Mzfp1^wt^-HA protein is strongly enriched in heterochromatin. It is most likely that most of Mzfp1 binds to heterochromatin, thereby reducing the efficiency of binding of this protein to the promoters of housekeeping genes that form interbands on polytene chromosomes (126).

Deletion of the MADF1 domain or CP190 interacting region did not noticeably alter the patterns of binding of the mutant Mzfp1-HA variants to polytene chromosomes compared to the Mzfp1^wt^-HA protein (Figure 4). On the contrary, deletion of the MADF2 domain led to a redistribution of the Mzfp1^ΔM2^-HA protein from pericentromeric heterochromatin to euchromatic regions: a large number of new or more intensely stained sites appear in the interbands. Thus, inability of Mzfp1^ΔM2^-HA to bind efficiently to heterochromatin increases its concentration in interbands of polytene chromosomes.

The Mzfp1^ΔZF^-HA mutant in which the 3–6 zinc fingers of the C2H2 cluster were deleted still bound strongly to several euchromatin sites and was highly enriched in the heterochromatin. It is possible that Mzfp1^ΔZF^-HA can be recruited to the chromatin via protein-protein interactions. Alternatively, MADF2 may be responsible for Mzfp1^ΔZF^-HA binding when the C2H2 cluster is deleted. To determine the potential roles of the MADF domains, we expressed *in vitro* the MADF1-MADF2 region (100–434 aa) and the C2H2 cluster (404–587 aa) fused with MBP. In EMSA (Supplementary Figure S16), only the C2H2 cluster specifically bound to four Mzfp1 motifs (Mzfp1^×4^). We confirmed the key role of the C2H2 cluster in specific binding to Mzfp1^×4^ via a luciferase reporter assay in S2 cells (Supplementary Figure S17). Although we have not been able to show specific binding of MADF2 to Mzfp1^×4^ DNA *in vitro*, MADF2 may specifically bind to unidentified sites.

Taken together, the results suggest that the MADF2 domain may be responsible for the recruitment of Mzfp1 to the heterochromatin. The CP190 interacting region and both MADF domains are required for Mzfp1 functionality.

### Testing the binding of Mzfp1 variants in adult flies

**Figure 5.**
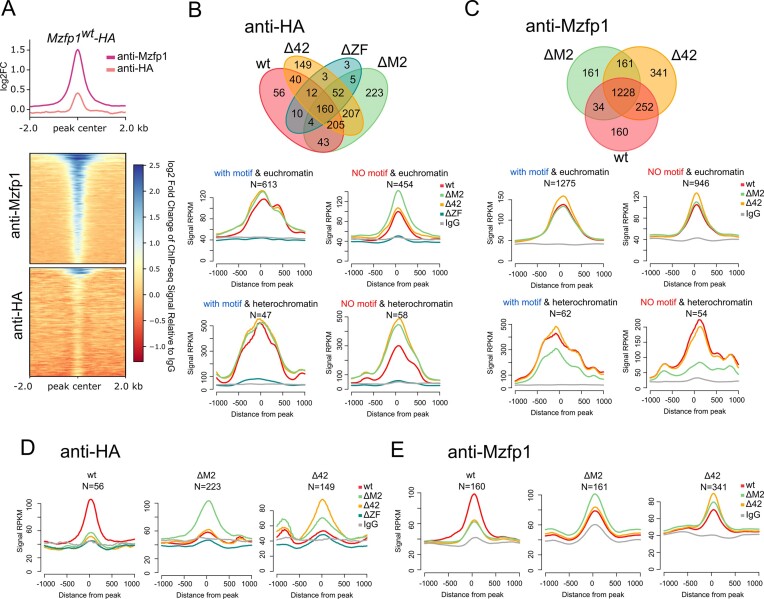
Binding specificity of Mzfp1 variants in adult flies. (**A**) Comparison of the Mzfp1 ChIP-Seq signal (log_2_(RPKM)) in adult Mzfp1^wt^-HA flies with anti-Mzfp1 and anti-HA antibodies at the combined set of binding sites identified by these antibodies. (**B**) Colocolization of Mzfp1 in Mzfp1^wt^-HA (wt), Mzfp1^ΔM2^-HA (ΔM2), Mzfp1^Δ42^-HA (Δ42), and Mzfp1^ΔZF^-HA (ΔZF) lines (on the top) and average ChIP-Seq signals (on the bottom) for wt, ΔM2, Δ42, ΔZF, and the pre-immune control (IgG) in the combined set of wt, ΔM2, Δ42 and ΔZF binding sites with and without the motif located in euchromatic and heterochromatic regions with anti-HA antibodies. (**C**) Colocolization of Mzfp1 in wt, ΔM2, Δ42 lines (on the top) and average ChIP-Seq signals (on the bottom) for Mzfp1 in wt, ΔM2 and Δ42 lines, and IgG in the combined set of wt, ΔM2 and Δ42 binding sites with and without the motif located in euchromatic and heterochromatic regions with anti-Mzfp1 antibodies. (**D**) Average ChIP-Seq signals for Mzfp1 in wt, ΔM2, Δ42 and ΔZF lines, and IgG in binding sites enriched in wt, ΔM2, Δ42 and ΔZF lines obtained with anti-HA antibodies. For the analysis with anti-HA antibodies only binding sites intersecting with ones identified in Mzfp1^wt^-HA (anti-HA) lines, *y^1^w^118^* adults or embryos were considered in this analysis. (**E**) Average ChIP-Seq signals for Mzfp1 in wt, ΔM2 and Δ42 lines, and IgG in binding sites enriched in wt, ΔM2 and Δ42 lines obtained using anti-Mzfp1 antibodies.

To further understand the role of Mzfp1 domains in specific recruitment to genomic regions, we compared the distribution of Mzfp1 variants targeted with 3×HA-epitope in two- or three-day-old adult flies (CG1603^attP^/CyO; U:Mzfp1*/TM6,Tb) using a ChIP-seq approach. Chromatin immunoprecipitation followed by next-generation sequencing was performed on chromatin extracted from the *Mzfp1^wt^, Mzfp1^ΔM2^, Mzfp1^Δ42^* and *Mzfp1^ΔZF^* adults, with two independent biological replicates using rat anti-Mzfp1 antibodies and antibodies against the HA-epitope (Figure 5A). As a control, ChIP-seq was also performed on chromatin extracted from *y^1^w^1118^*adults in two independent biological replicates using rat anti-Mzfp1 antibodies.

We first compared Mzfp1 binding in embryos and adult 2–3 day-old flies of the *y^1^w^1118^*line (Supplementary Figure S18). In total, 974 peaks with the motif and 692 peaks without the motif were found in the Mzfp1 samples from adult flies. Thus, Mzfp1 is identified significantly better in adult flies than in embryos, which may be a consequence of less accessibility of the Mzfp1 epitope to antibodies at the embryonic stage. However, comparison of Mzfp1 enrichment at embryonic and adult genomic sites revealed that Mzfp1 binds to the same regulatory elements at both stages of *Drosophila* development (Supplementary Figure S18). Summing up the binding sites at the embryonic and adult stages allowed us to increase the total number of the Mzfp1 binding sites with motif from 390 (Figure 2) to 966 (Supplementary Figure S19), most of which are located in gene promoters. With an increase in the number of Mzfp1 sites, high colocalization of Mzfp1 binding sites with BEAF-32 (78%), with CP190 (82%) and with Pita (18%) remains.

Next, we compared Mzfp1 binding in adults 2–3 day-old flies of *y^1^w^1118^*and *CG1603^attP^/CyO; U:Mzfp1^WT^/ TM6,Tb* (*Mzfp1^wt^*) (Supplementary Figure S20). Despite several times higher Mzfp1 expression in the *Mzfp1^wt^*line relative to the *y^1^w^1118^* line, very similar Mzfp1 binding efficiency and specificity is observed in adults from both lines. In ChIP-seq with anti-HA antibodies, the same but less intensive Mzfp1 peaks were identified in *Mzfp1^wt^* adults (Figure 5A). The greater effectiveness of antibodies against Mzfp1 compared to antibodies against HA can be explained by the fact that only the part of Mzfp1 associated with endogenous sites contains the HA-epitope in *Mzfp1^wt^* line.

At the end, we compared the binding strength of Mzfp1^wt^-HA, Mzfp1^ΔM2^-HA, Mzfp1^Δ42^-HA and Mzfp1^ΔZF^-HA with genomic sites in ChIP-seq datasets generated with anti-HA antibodies (Figure 5B). In contrast to Mzfp1^wt^-HA, we did not observe true peaks or any significant signals for Mzfp1^ΔZF^-HA, confirming that the C-terminal C2H2 cluster was critical for specific binding of Mzfp1 to chromatin. The high content of Mzfp1^ΔZF^-HA on the pericentromeric heterochromatin of polytene chromosomes (Figure 4) can be explained by the interaction of Mzfp1^ΔZF^-HA with heterochromatin proteins without direct binding to DNA.

ChIP-seq datasets with anti-HA (Figure 5B) and anti-Mzfp1 (Figure 5C) antibodies showed that, basically, Mzfp1^wt^-HA, Mzfp1^ΔM2^-HA and Mzfp1^Δ42^-HA bind to chromatin sites in euchromatin regions with the similar efficiencies. However, there were groups of sites that bound only one of the analyzed protein variants effectively (Figure 5D, E and Supplementary Figure S21A). These peaks were predominantly associated with euchromatin promoters (Supplementary Figure S21B–D).

In ChIP-seq data with anti-HA antibodies, we did not see significant difference in binding of Mzfp1^wt^-HA, Mzfp1^ΔM2^-HA and Mzfp1^Δ42^-HA to heterochromatin sites (Figure 5B). At the same time, ChIP-seq data with anti-Mzfp1 antibodies showed considerable decreasing of Mzfp1 binding to heterochromatin regions in*Mzfp1^ΔM2^* adults (Figure 5C).

Overall, our ChIP-seq analysis provides further insight into the role of Mzfp1 domains in specific recruitment to genomic regions. The results suggest that the C-terminal C2H2 cluster is critical for the specific binding of Mzfp1 to chromatin, while the CP190 interacting region and the MADF2 domain bind to different partner proteins and determine the specificity of Mzfp1 association with different regulatory regions.

### Mzfp1, Pita and Su(Hw) cooperate in boundary activity of the proximal part of the *Fub* boundary

**Figure 6.**
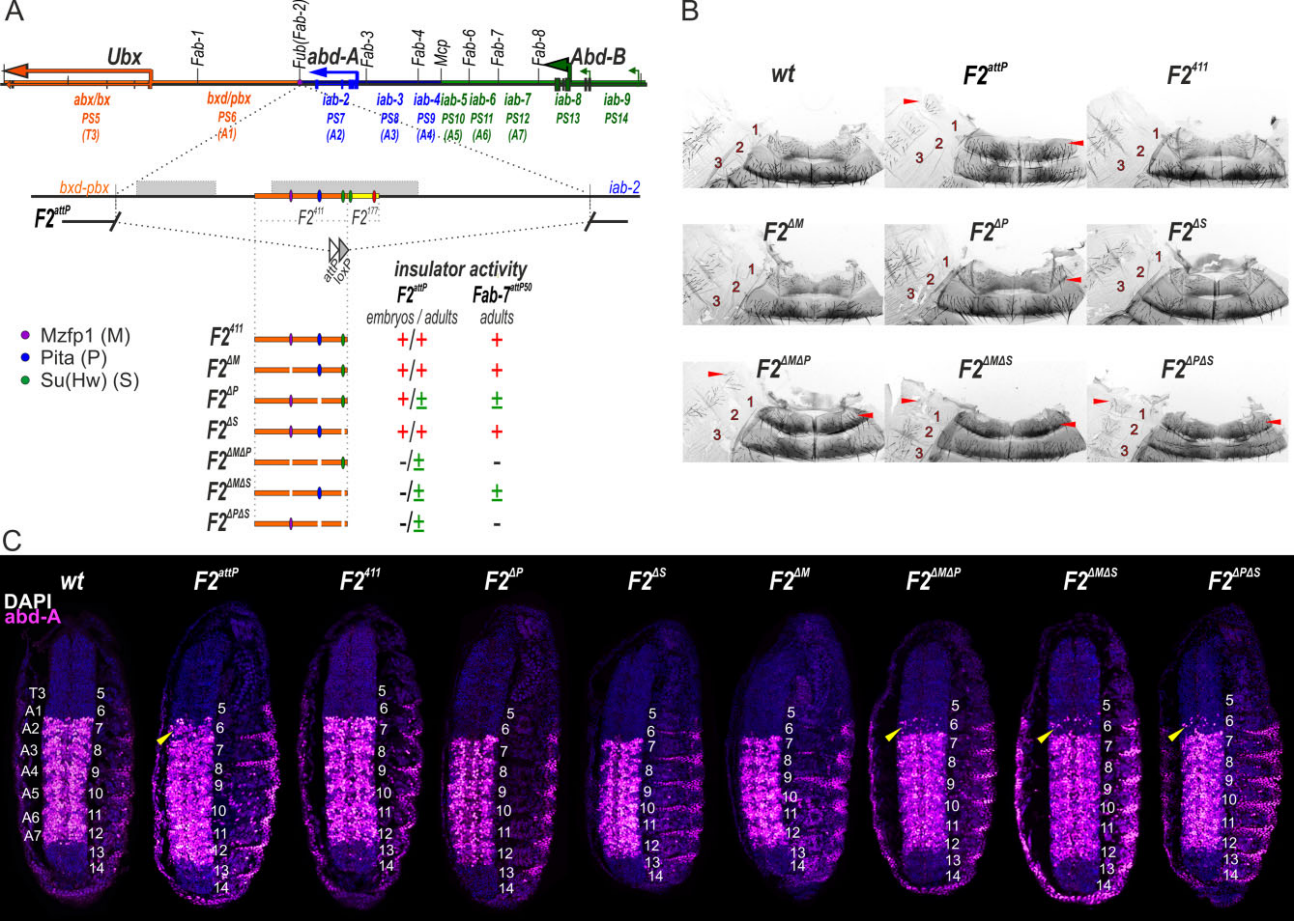
Analysis of the functional roles of Mzfp1, Su(Hw), and Pita in the activity of *F2^411^* inserted instead of the full *Fub* boundary. (**A**) Schematic representation of the BX-C including *Ubx*, *abd-A*, and *Abd-B* genes and the *Fub* boundary. The *Ubx*, *abd-A* and *Abd-B* genes are shown in orange, blue, and green horizontal arrows, respectively. Their regulatory regions are marked with the same color-coded bars. The boundaries (*Fab-1*, *Fub (Fab-2)*, *Fab-3*, *Fab-4, Mcp*, *Fab-6*, *Fab-7* and *Fab-8*) that separate segment-specific regulatory domains (*abx/bx, bxd/pbx* – regulate *Ubx*, *iab-2*, *iab-3* and *iab-4* – *abd-A* and *iab-5*, *iab-6*, *iab-7* and *iab-8,9* – *Abd-B*) are indicated by vertical black bars. A schematic of the *F2^attP^* deletion is shown below. The *Fub* boundary has two hypersensitive regions (gray boxes). *F2^411^* is shown as an orange bar with marked Mzfp1 (magenta oval), Pita (blue oval), and Su(Hw) (green oval) binding sites. *F2^177^* is shown as an yellow bar with marked Su(Hw) (green oval) and dCTCF (red oval) binding sites. Mutated sites are shown as an erased region in place of the corresponding binding sites. The endpoints of the *F2^attP^* deletion are indicated by breaks in the black lines. The *F2^411^*fragments used in the experiments and the results of deletion and rescue are shown at the bottom: (+) – the fragment has insulator activity in embryos/adults; A1(PS6) and A2(PS7) segments have the *wt* phenotype; (-) the fragment has no insulator activity; A1(PS6) transforms into A2(PS7); (±) – the fragment has partial insulator activity in some cells; A1(PS6) has several characteristics of the A2(PS7). (**B**) Morphology of the A1 and A2 abdominal segments (numbered) under different *F2^attP^* replacement schemes. In *wt* the A1 sternite is absent; the tergite has a specific form and is thinned towards the center, with no long bristles. A2 comprises a quadrilateral sternite with vertically oriented bristles and a tergite with a pigmented stripe and long bristles. In the *F2^attP^* deletion, the A1 tergite is wider; its posterior margin is pigmented and covered in large bristles as in the A2; the A1 sternite is covered in bristles and resembles the sternite in A2. The signs of the A1→A2 transformation are indicated by red arrowheads. (**C**) Expression of the *abd-A* gene in embryos with different *F2^attP^* replacements. Each panel shows a confocal image of the embryo at stage 14 stained with antibodies against the Abd-A protein (magenta). The parasegments are numbered from 5 to 14 on the right side of each embryo image. The number of the approximately corresponding adult segment is indicated on the left. In *wt* embryos, *abd-A* is inactive in PS5 and PS6, whereas it is active in PS7-PS12. In *F2^attP^* embryos, Abd-A was detected in PS6. The yellow arrowheads indicate the ectopic expression of *abd-A*.

A single Mzfp1 peak with a motif was identified in the proximal part of the *Fub* (*Front-ultraabdominal*) boundary (Figure 6A). This boundary restricts the *bithoraxoid/postbithoraxoid* (*bxd/pbx*) domain, which activates the *Ubx* gene in the parasegment 6 (PS6) in embryos (A1 segment in adults), from the *infraabdominal-2* (*iab-2*) domain, which controls *abd-A* in PS7 (A2) in the BX-C (63) (Figure 6A). Deletions of the *Fub* boundary results in ectopic activation of *abd-A* by the *bxd* enhancers in the A1 segment (63), leading to the transformation of the A1 segment to A2. Previously, the 2106 bp *Fub* boundary was replaced by the *attP* site in the *F2^attP^* deletion (57). *F2^attP^* homozygotes, in addition to displaying strong phenotype changes, demonstrated low viability and sterility (Figure 6B, (58)). In *F2^attP^* embryos, *abd-A* expression observed in PS6, reflecting premature activation of the *iab-2* enhancers by the *bxd/pbx* domain (Figure 6C). A previous study showed that the distal 177 bp fragment of *Fub* with binding sites for dCTCF and Su(Hw) can effectively function as boundary, rescuing the 2106 bp *F2^attP^* deletion of the *Fub* boundary (Figure 6A) (35,57). Here, we tested the 411 bp proximal part of *Fub, F2^411^*, that contains motifs not only for Mzfp1 but also for the architectural proteins Pita and Su(Hw). Using EMSA, we confirmed (Supplementary Figure S22) the binding of Pita, Su(Hw), and Mzfp1 with corresponding binding sites in *F2^411^*. Deletion of individual binding sites resulted in the loss of protein binding to its motif (Supplementary Figure S22). Integration of *F2^411^*into *F2^attP^* completely restores the *wt* phenotype, suggesting that the proximal 411 bp region can substitute the *Fub* boundary (Figure 6B). No *abd-A* expression in PS6 was observed in embryos (Figure 6C), confirming that *F2^411^*functions at all developmental stages.

**Figure 7.**
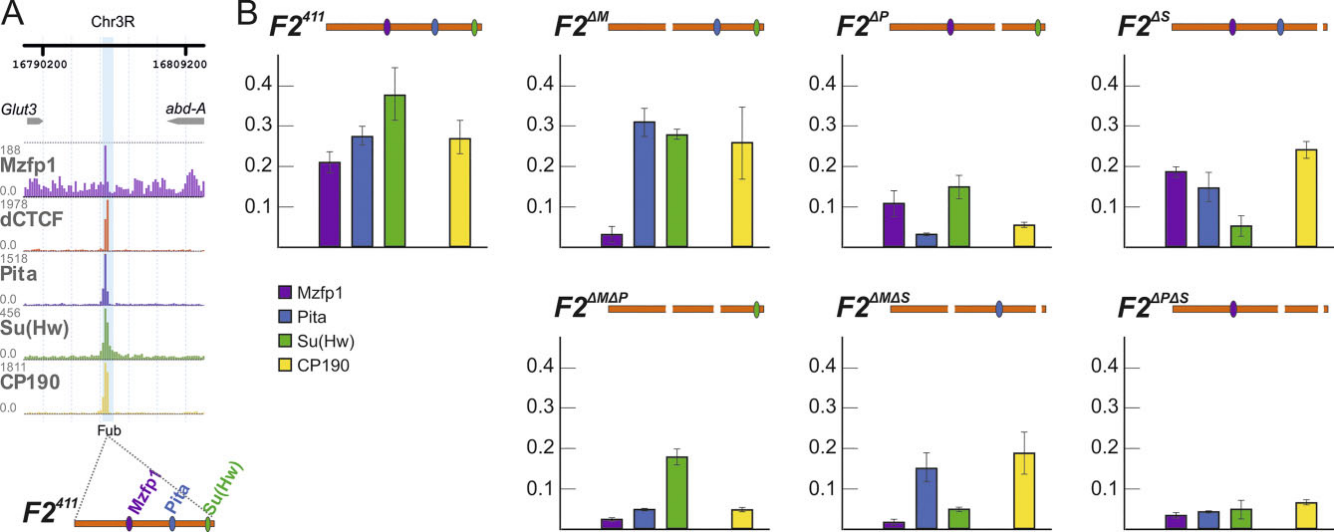
Binding of Mzfp1, Su(Hw), and Pita to *F2^411^* and mutant derivatives in two-day-old adult males. (**A**) Tracks for Mzfp1, dCTCF, Pita, Su(Hw), and CP190 binding profiles at the *Fub*boundary via a ChIP-Seq analysis using embryos. (**B**) Binding of Mzfp1 (magenta), Pita (dark blue), Su(Hw) (green), and CP190 (yellow) with *F2^411^*, *F2^ΔM^*, *F2^ΔP^*, *F2^ΔS^*, *F2^ΔMΔP^*, *F2^ΔMΔS^* and *F2^ΔPΔS^* elements. The results of ChIPs are presented as the percentage of input DNA, normalized against a positive genomic site: *62D*, for Su(Hw), *50E*, for Pita and *94C*, for Mzfp1 and CP190 binding. The error bars indicate SDs of quadruplicate PCR measurements from three independent biological samples of chromatin.

We then investigated the contribution of proteins to the *F2^411^* boundary activity by introducing single and double deletions of their sites. Deletion of the Su(Hw) (*F2^ΔS^*) or Mzfp1 (*F2^ΔM^*) sites did not affect the boundary activity of *F2^411^* (Figure 6B). However, deletion of the Pita site led to a moderate wing phenotype of the *F2^ΔP^* flies. Simultaneous deletion of sites for any two of the proteins strongly affected the boundary function of *F2^411^*. The flies displayed a strong transformation of A1 into A2. However, neither viability nor fertility was significantly decreased. This was correlated with moderate activation of *abd-A* in PS6 (Figure 6C). ChIP-Seq profiles demonstrated well-defined peaks for Mzfp1, Pita, Su(Hw), dCTCF, and CP190 proteins in embryos at the *Fub* boundary (Figure 7A). To further investigate their binding, we performed a qChIP assay with chromatin from three-day-old adult flies. Our analysis examined Mzfp1, Pita, Su(Hw), and CP190 binding with *F2^411^, F2^ΔM^, F2^ΔP^*, F2^ΔS^, *F2^ΔMΔP^*, *F2^ΔMΔS^* and *F2^ΔPΔS^*. The results confirmed that Mzfp1, Pita, Su(Hw), and CP190 were able to bind to the *F2^411^* region *in vivo* (Figure 7B). Inactivation of the Mzfp1 motif did not significantly affect Su(Hw) or Pita binding (Figure 7B, Supplementary Figure S23). However, we found that Pita and Su(Hw) binding was interdependent, indicating that these proteins cooperatively bind to the insulator. Inactivation of either the Su(Hw) or Pita motif affected Mzfp1 binding. Simultaneous mutation of both Su(Hw) and Pita motifs almost completely reduced the association of Mzfp1 with the *F2^411^*insulator. Similarly, simultaneous inactivation of Pita and Mzfp1 motifs abolished Su(Hw) recruitment to *F2^411^*.

CP190 recruitment was highly dependent on Pita binding but not on the presence of Su(Hw) or Mzfp1 on the insulator (Figure 7B, Supplementary Figure S23). Even double inactivation of Mzfp1 and Su(Hw) motifs only partially decreased the binding of CP190 to the mutant insulator. These results explained why inactivation of Pita had a more pronounced effect on the boundary activity compared to mutation of the Su(Hw) or Mzfp1 motifs.

To confirm our results, we used another model system that allowed for estimating the insulator function of the *F2^411^*boundary (Supplementary Figure S24). In the *Fab-7^attP50^* platform, the *Fab-7* boundary was replaced by an *attP* site (127). The *Fab-7* boundary blocks interaction between regulatory elements in the *iab-6* and *iab-7* domains, thereby stimulating the correct expression of *Abd-B* in the PS11 and PS12 parasegments, respectively. Deletion of the *Fab-7* boundary fuses the *iab-6* and *iab-7* regulatory domains, resulting in mutant flies exhibiting an exclusively gain-of-function (GOF) transformation of PS11 into PS12 (68,128–130). The GOF phenotype is a consequence of the *iab-6* initiator premature activating the *iab-7* domain in PS11 (A6), leading to the overexpression of *Abd-B* in this segment and morphological transformation A6 into A7 (Supplementary Figure S24).

We investigated whether *F2^411^*could function as a boundary instead of the deleted *Fab-7* sequences. Phenotypic analysis of the cuticle of adult male *F2^411^* flies indicated that the 411 bp element completely blocked the crosstalk between *iab-6* and *iab-7* (Supplementary Figure S24). To examine the contributions of Mzfp1, Su(Hw), and Pita to the boundary function, we substituted *Fab-7* with mutant variants of *F2^411^* with the deletion of either one or two binding sites. As expected, deletion of the Mzfp1 or Su(Hw) site did not influence boundary activity, while deletion of the Pita site resulted in a moderate GOF phenotype, suggesting partial inactivation of the boundary function. Double mutation of Pita and either Mzfp1 or Su(Hw) almost completely eliminated the boundary activity. In contrast, deletion of the Su(Hw) and Mzfp1 sites resulted in only a moderate GOF phenotype. These results confirmed that the Pita binding site is the most critical for *F2^411^* activity, but Su(Hw) and Mzfp1 also contribute to the boundary function.

## Discussion

Here, we introduced a novel architectural protein, Mzfp1, that interacts with CP190, a known multifunctional protein involved in the recruitment of transcription complexes, the creation of open chromatin regions, distant interactions, and the activity of insulators. We found that two unstructured regions (152–194 aa, 292–320 aa) in Mzfp1 interact with the 210–245 aa and 309–390 aa of CP190. The previously characterized architectural C2H2 proteins M1BP, ZIPIC and Opbp bind to the same regions of CP190 (118). Like Mzfp1, these proteins are primarily involved in organizing the architecture of active housekeeping gene promoters (33,34,38,39). Our results suggest that the 161–194 aa and 294–319 aa regions of Mzfp1 cooperatively interact with CP190, as deletion of the 152–194 aa region led to a significant but not complete loss of interaction between mutant Mzfp1^Δ42^ and CP190 *in vivo*.

Mzfp1 has an unusual combination of two MADF domains and a C-terminal cluster of C2H2 domains. Mzfp1 is important for *Drosophila* development, and Mzfp1-like proteins are found not only in the genus *Drosophila* but also in distantly related species from the Schizophora section of Diptera, suggesting the functional importance of this group of proteins. MADF domains are responsible for specific DNA binding of several transcription factors (131). Many transcription activators contain MADF domains that have two activities: specific binding to DNA and interaction with transcription complexes (72,76,132).

Our deletion analysis suggests that the C-terminal cluster of C2H2 domains is responsible for the binding of Mzfp1 to highly specific long motifs in regulatory regions. Therefore, MADF domains primarily contribute to protein-protein interactions involving Mzfp1. Both MADF domains are essential for Mzfp1 activity. MADF1 is likely involved in the recruitment of transcription complexes, as was previously shown for the promoter-associated transcription factor Adf1 (76).

The MADF2 domain is required for the association of Mzfp1 with heterochromatin. It seems likely that MADF2 interacts with unknown heterochromatin proteins. The previously characterized MADF-containing protein Stwl, like Mzfp1, is associated with promoters located both in heterochromatic and euchromatic regions (77,133). Some MADF domains may acquire a specific function of attracting transcription factors to the heterochromatin compartment. However, it is possible that the interaction of MADF2 with DNA can stabilize the binding of Mzfp1 to some chromatin sites.

Mzfp1 preferentially binds to gene promoters located in both euchromatin and heterochromatin regions. BEAF-32 and Pita are the closest partners of Mzfp1, and these proteins can cooperate to form active promoters and co-recruit CP190. We recently found that mutant dCTCF and Pita proteins that had lost the ability to recruit CP190 did not affect the binding of CP190 to the promoters associated with dCTCF or Pita (46,87). Therefore, the results are consistent with the model hypothesis according to which various combinations of architectural proteins bind to promoters and co-recruit CP190 and possibly other transcriptional complexes. Our results showed that Mzfp1, along with Pita and Su(Hw), plays a role in the insulator function of the proximal part of the *Fub*boundary that splits the 300 kb BX-C into two TADs (70,134). In the cells of parasegment 6 (PS6), the *Fub* boundary lies between an active *bxd* domain and a silenced *iab-2* domain (63,64). When the *Fub* boundary is deleted, the *bxd/pbx* initiators inappropriately activate *iab-2* in PS6 (63). The *Fub* boundary consists of a functionally redundant 411 bp proximal part characterized in this work (*Fub^411^*) and the previously described 177 bp distal part (*Fub^177^*). The *Fub^411^* region can function as an efficient insulator when it is substituted for the endogenous *Fub* or the *Fab-7* boundary (135). *Fub^411^* contains motifs for Su(Hw), Pita, and Mzfp1. Only the deletion of the Pita motif strongly affected the boundary function of *Fub^411^*. The presence of Pita is required for the binding of Su(Hw) and Mzfp1, while the recruitment of Pita to *Fub^411^*is only partially dependent on the presence of Su(Hw). Even the inactivation of Mzfp1 and Su(Hw) motifs simultaneously did not significantly reduce the binding of Pita to *Fub^411^*. Previously, we found that the binding of Pita to the 340 bp *Mcp*boundary did not depend on the adjacent dCTCF motif (56,58). In contrast, the binding of dCTCF depends on the presence of the Pita motif in *Mcp*. Thus, Pita can be considered as one of the organizer proteins that facilitates the binding of other C2H2 architectural proteins to the boundaries in BX-C. This correlates with the main contribution of Pita in recruiting to *Fub^411^* of CP190 that is essential for the insulator activity of boundaries (46,53,54). Since even two Pita motifs are insufficient to form a functional insulator (136), it seems likely that additional, yet unknown, architectural C2H2 proteins are associated with *Fub^411^*.

Taken together, our results indicate that Mzfp1 partners with the M1BP and Pita proteins in the formation of active promoters, and that it can cooperate with Pita and Su(Hw) in the formation of insulators. Since the same architectural C2H2 proteins can form both promoters and boundaries/insulators, this has perhaps clarified why promoters can function similarly to insulators, as shown in several recent (40,53,137) and previous (138,139) studies.

## Supplementary Material

gkae393_Supplemental_Files

## Data Availability

NGS data have been deposited at the NCBI Gene Expression Omnibus (GEO) under accession numbers GSE242677 and GSE242679.
