# Supplementary material for: New Drosophila promoter-associated architectural protein Mzfp1 interacts with CP190 and is required for housekeeping gene expression and insulator activity
Source: Nucleic Acids Res. 2024 May 20;52(12):6886–905. doi: 10.1093/nar/gkae393 (PMC11229372; doi:10.1093/nar/gkae393)
Supplement: gkae393_Supplemental_Files [file gkae393_supplemental_files.zip › Sokolov etal_SupplM_0605.pdf]

Supplementary Materials

New Drosophila promoter-associated architectural protein Mzfp1 interacts with CP190 and is required for housekeeping gene expression and insulator activity

Vladimir Sokolov, Olga Kyrchanova, Natalia Klimenko, Anna Fedotova, Airat Ibragimov, Oksana Maksimenko and Pavel Georgiev

Supplementary Tables

Supplementary Table S1. The list of oligonucleotides used in the work.

Supplementary Table S2. Genes whose promoters overlap with Mzfp1 binding sites.

Supplementary Table S3. Results of gene ontology (GO) enrichment analysis (overrepresentation analysis) for 3 sets of Mzfp1 binding sites in euchromatin: with motif (N=390), without motif (N=358) and all sites (N=748). GO enrichment analysis for the sites located in heterochromatin did not reveal significant results.

Supplementary Table S4. Results of differential expression analysis in $CG1603^{attP}/CG1603^{attP}$ larvae (compared to wild type) for genes containing the Mzfp1 binding site in the promoter region (mo.het – with motif and located in heterochromatin, nomo.het – without motif and located in heterochromatin, mo.eu – with motif and located in euchromatin, nomo.eu – without motif and located in euchromatin).

Supplementary Figures

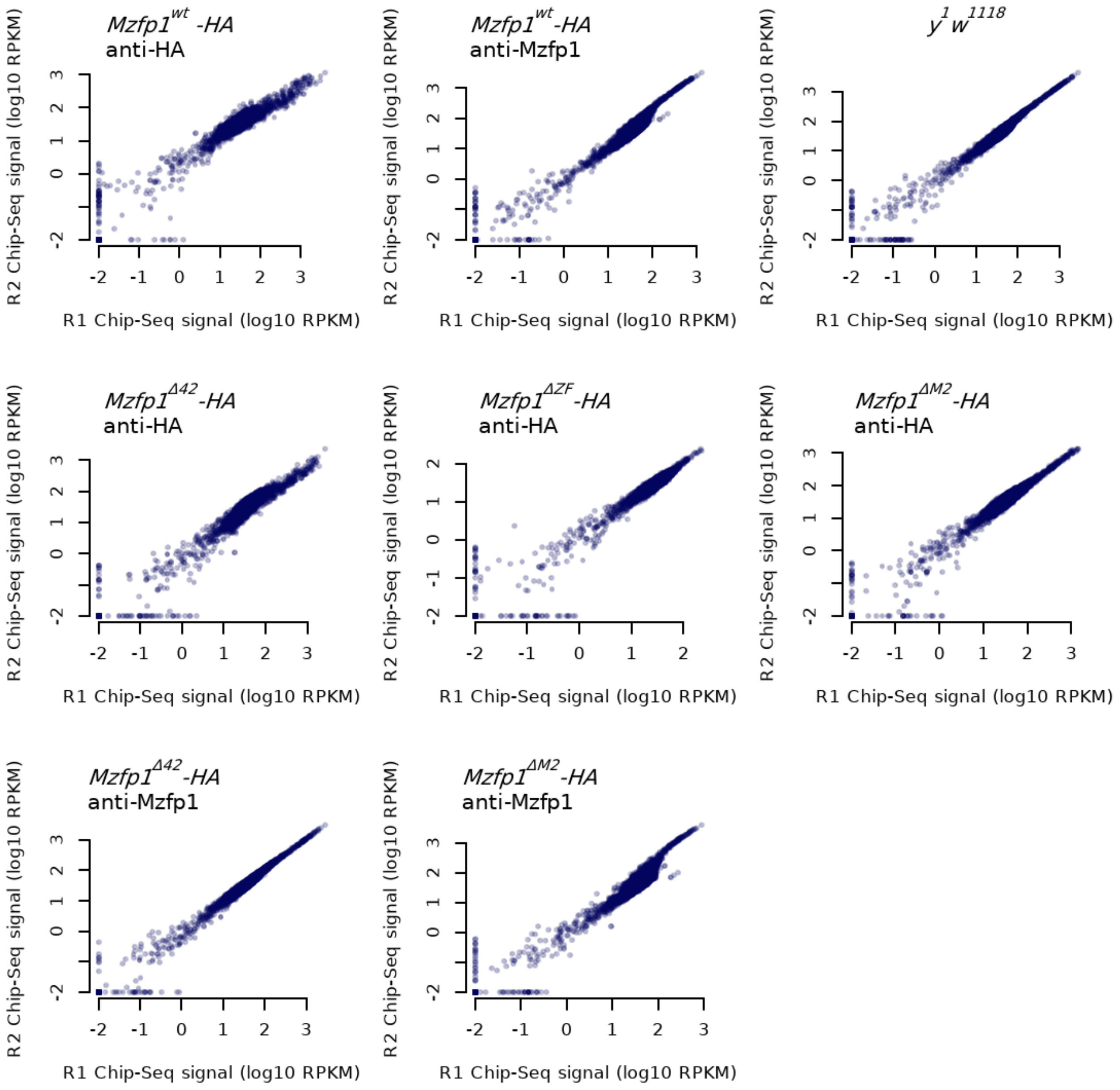

Figure S1. Correlation plots showing reproducibility between ChIP-seq biological replicates. Values represent log10 transformed RPKM values in the promoters of 17793 genes.

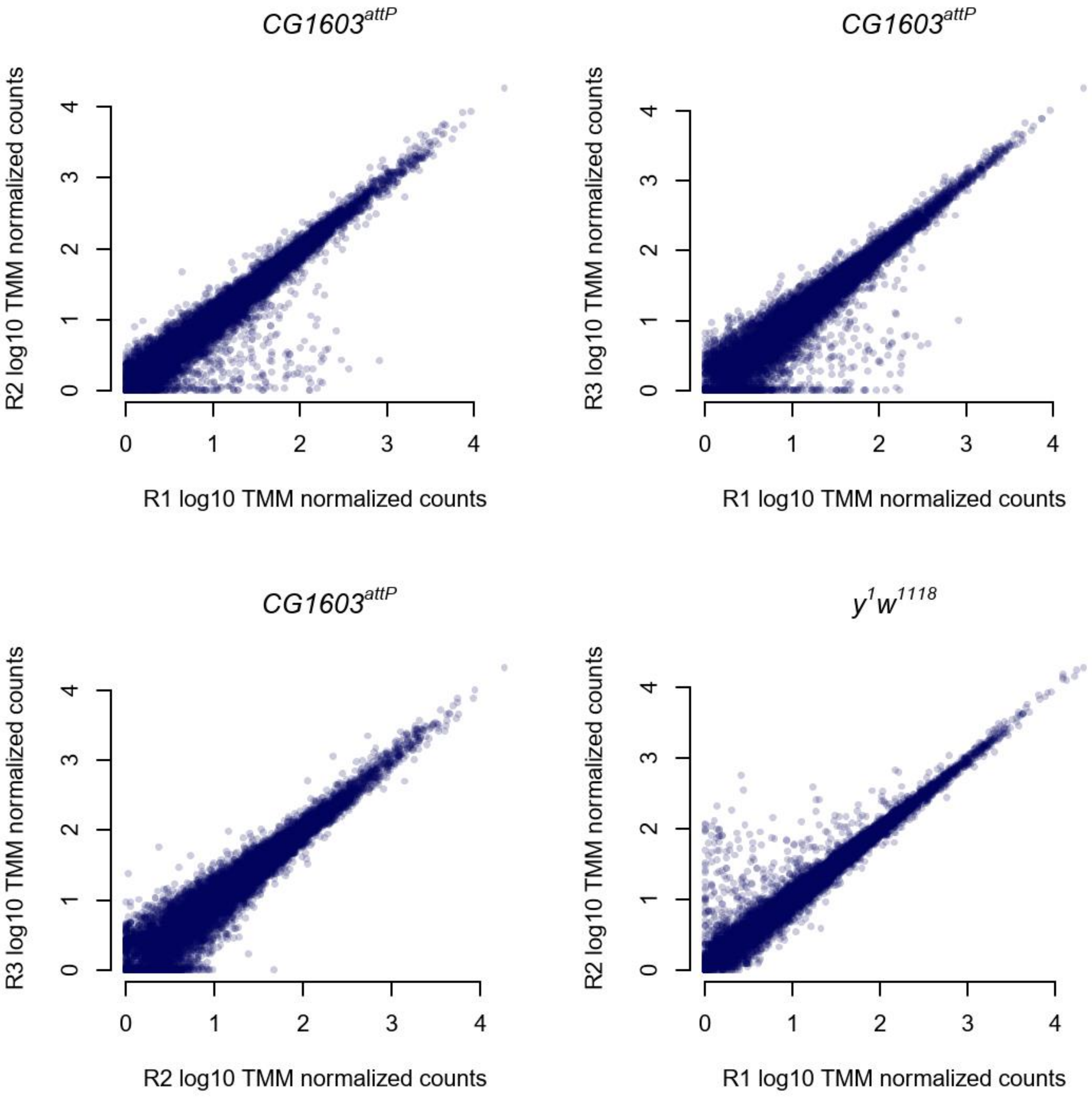

Figure S2. Correlation plots showing reproducibility between RNA-seq biological replicates. Values represent TMM normalized counts after log10 transformation for 11434 genes.

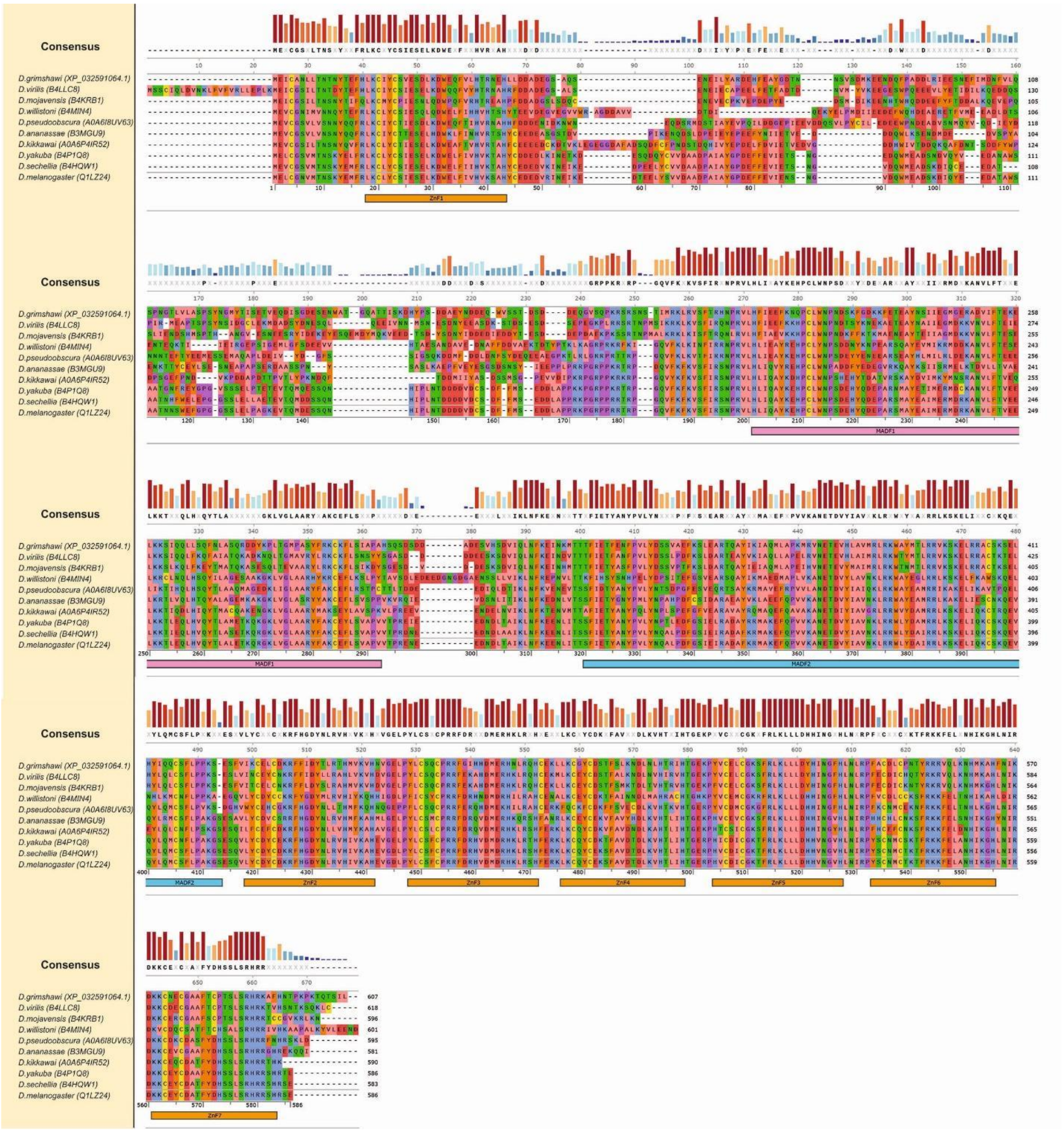

Figure S3. Multiple sequence alignment (Clustal Omega algorithm) of the orthologous Mzfp1 among species of *Drosophila* from different subgroups demonstrated a high degree of homology between the Mzfp1 proteins. The levels of homology for particular amino acid residues are indicated above the consensus sequence as colored bars: from brown with 100% similarity to dark blue with less than 10% similarity and grey with 'no homology'. Amino acid residues are marked with the Zappo color scheme in accordance with their physico-chemical properties (Aliphatic/hydrophobic ILVAM - pink; Aromatic FWY - orange; Positive KRH - dark blue; Negative DE - red; Hydrophilic STNQ - green; conformationally special PG - magenta; Cysteine - yellow). The numbers indicate the positions of amino acid residues. Domains of Mzfp1 are shown below the alignment as orange (C2H2 domains) and pink and blue (MADF domains) blocks.

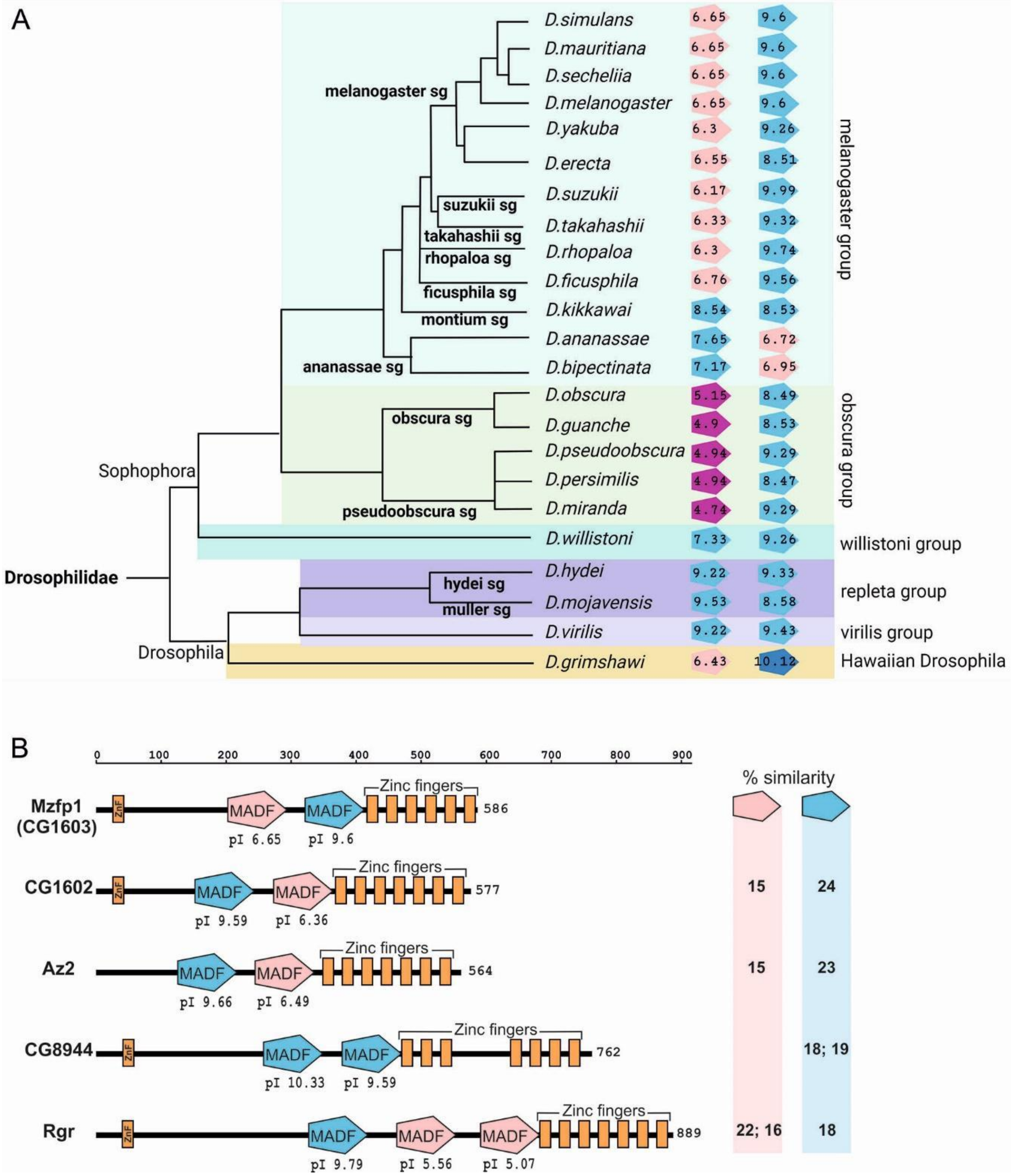

Figure S4. **(A)** Phylogenetic tree for species of Drosophilidae for which an Mzfp1 homologous protein has been found. The right panel indicates MADF domains that are contained in annotated proteins along with their isoelectric points. The colors of the pentagons indicate the MADF domains by their charges: blue = positively charged; pink/magenta = negatively charged. **(B)** The tandem organization of MADF domains in combination with the C2H2 domain cluster of Mzfp1 in paralogous proteins of *Drosophila melanogaster*. The right panel indicates the percentage of similarity between MADF domains (determined with the Clustal Omega algorithm). Created with BioRender.com.

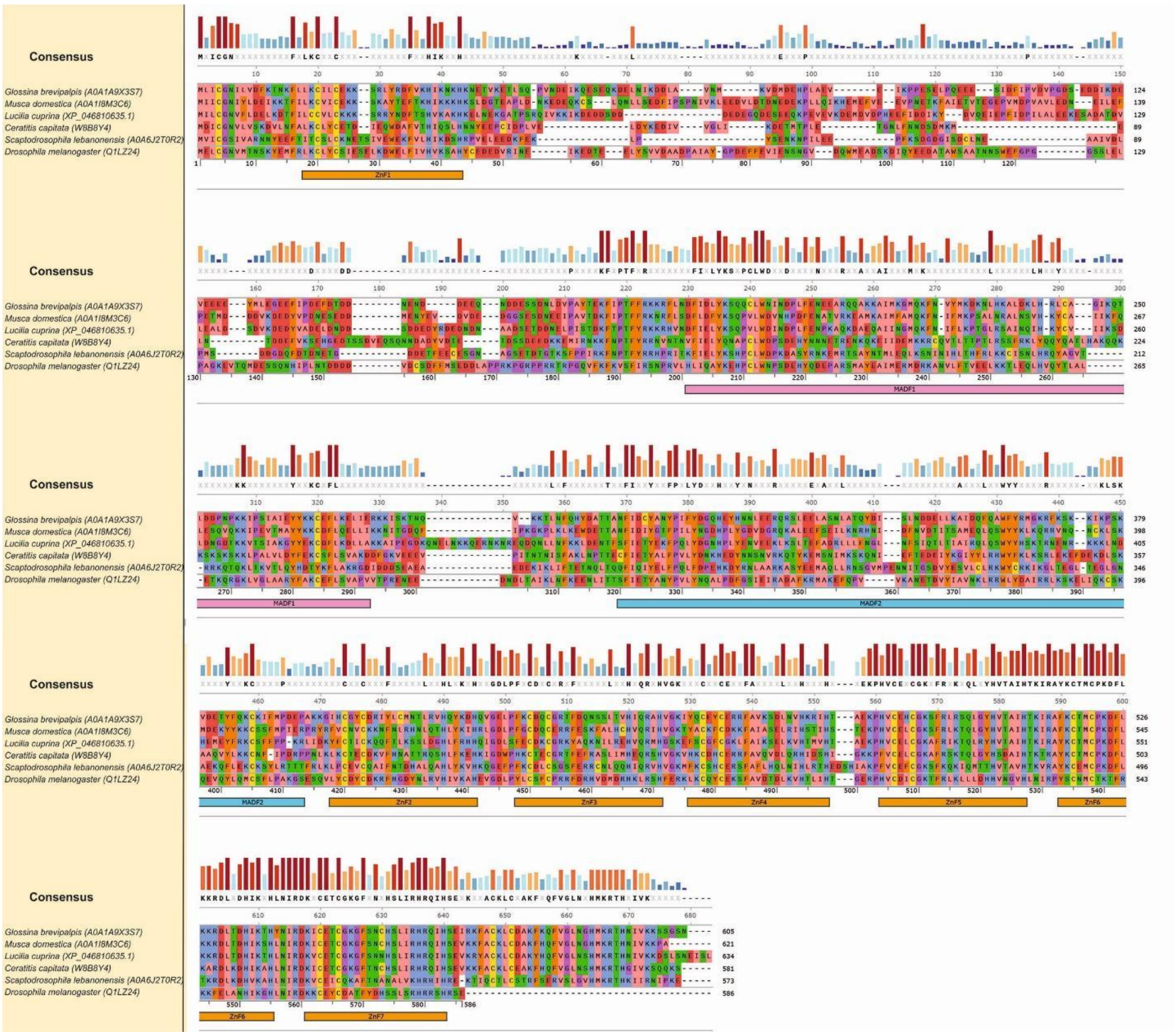

Figure S5. Multiple sequence alignment (Clustal Omega algorithm) of the orthologous Mzfp1 proteins among species from the Schizophora section of Diptera demonstrated low homology but maintained a common organization between the annotated proteins. The levels of homology for particular amino acid residues are indicated above the consensus sequence as colored bars: from brown with 100% similarity to dark blue with less than 10% similarity and grey with 'no homology'. Amino acid residues are marked with the Zappo color scheme in accordance with their physico-chemical properties (Aliphatic/hydrophobic ILVAM - pink; Aromatic FWY - orange; Positive KRH – dark blue; Negative DE - red; Hydrophilic STNQ - green; conformationally special PG - magenta; Cysteine - yellow). The numbers indicate the positions of amino acid residues. The domains of Mzfp1 are shown below the alignment as orange (C2H2 domains) and pink and blue (MADF domains) blocks.

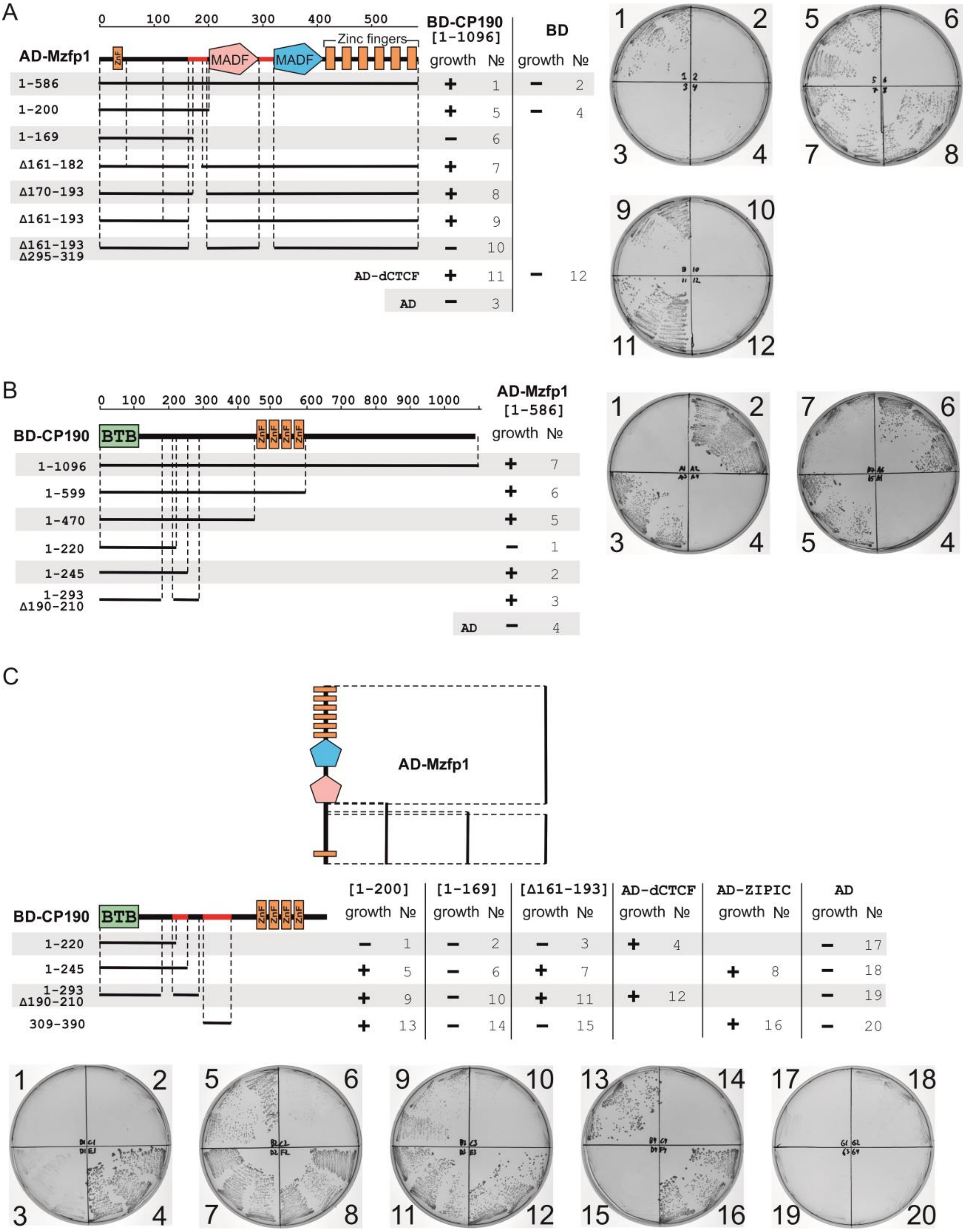

Figure S6. Results of Y2H assay. **(A)** Mapping of the Mzfp1 regions interacting with CP190. **(B)** Mapping of the CP190 domains interacting with Mzfp1. **(C)** Mapping of the Mzfp1- and CP190-interacting regions. The diagrams represent the domain organization of the Mzfp1 and CP190 proteins. Different fragments of CP190 were fused to the GAL4 DNA-binding domain (BD) and tested for interaction with fragments of Mzfp1 fused to the GAL4 activating domain (AD). All CP190 fragments were tested for the absence of interaction with the GAL4 activating domain alone (AD). Mzfp1 was tested for the absence of interaction with the GAL4 DNA-binding domain alone (BD). dCTCF and ZIPIC

fused with the GAL4 activating domain were used as positive controls that interact with CP190. The results are summarized in the columns, with the '+' and '–' signs referring to the presence and absence of interaction, respectively. The yeast growth after co-transformation with corresponding plasmids is shown on the photo of Petri dish. Numbers in the columns and at the photo of Petri dish indicate corresponding sector. Potential regions in Mzfp1 that interact with CP190 are indicated by red lines.

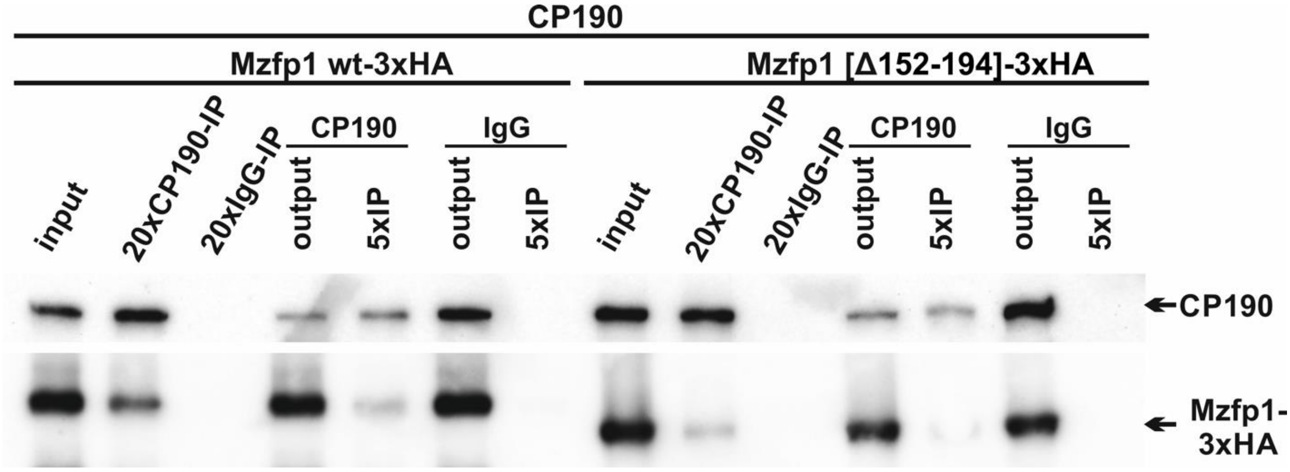

Figure S7. Total extracts from *Drosophila* S2 cells co-transfected with CP190 and Mzfp1-3xHA were immunoprecipitated with antibodies against CP190 or nonspecific IgG as a negative control, and the immunoprecipitates (IP) were analyzed by Western blotting for the presence of HA-tagged proteins. Inputs show the starting samples of extract, outputs are supernatant after sedimentation of immunoprecipitated material. CP190 and IgG immunoprecipitates are shown concentrated relative to the input by factors of 5 and 20.

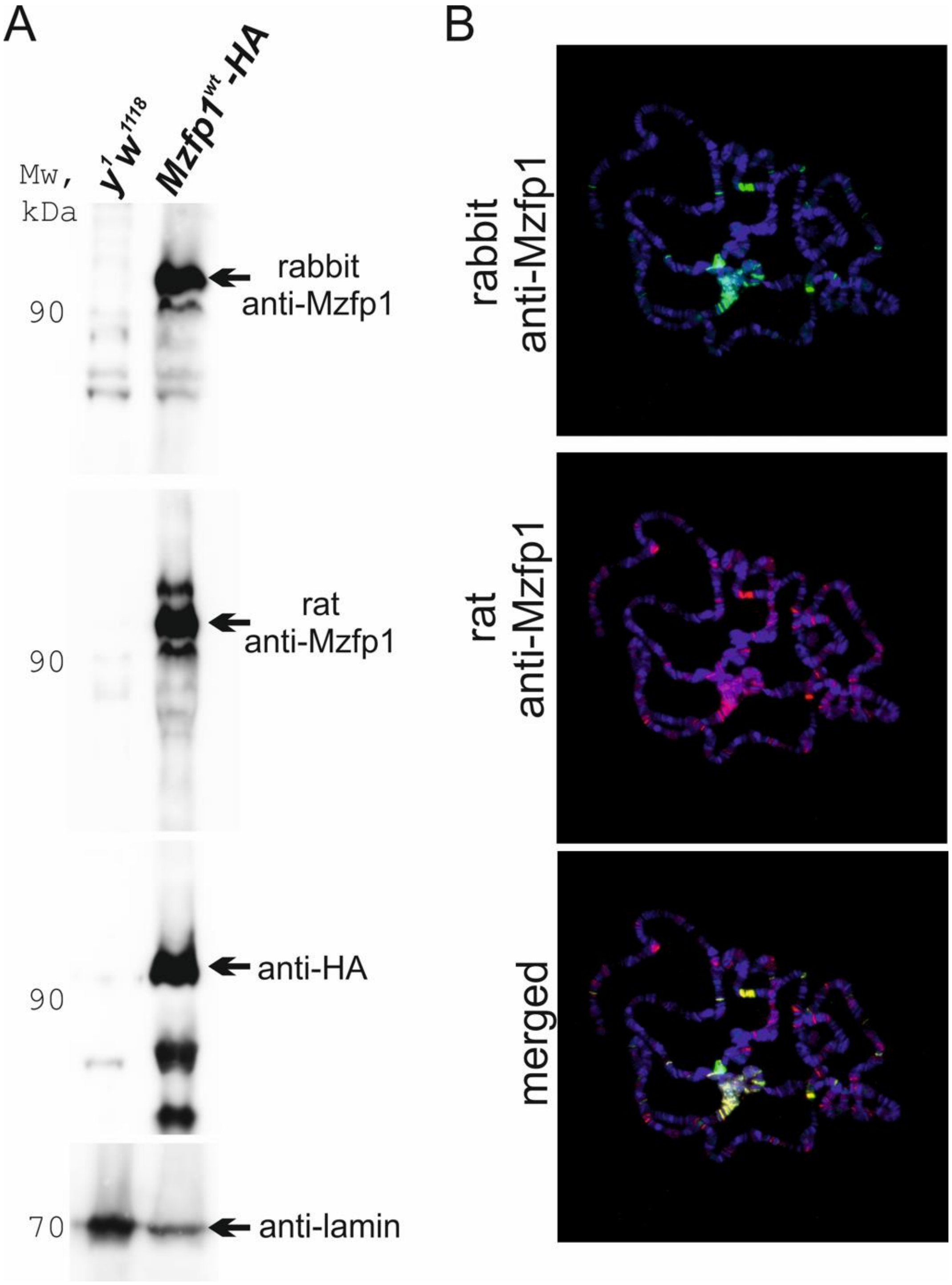

Figure S8. (A) Immunoblot analysis (6% SDS PAGE) of protein extracts prepared from adult two-day-old males (the *y¹w¹¹¹⁸* line and the *CG1603^{attP}/CyO;U:Mzfp1^{wt}-HA/TM6,Tb* line expressing Mzfp1^{wt}-HA) with rabbit and rat antibodies against Mzfp1 protein and the HA-epitope. Antibodies for lamin Dm0 were used as an internal control. **(B)** Distribution of Mzfp1 on the polytene chromosomes from third-day female larvae from the *Oregon* line. The panels show the immunostaining of proteins using rabbit (green) and rat (red) anti-Mzfp1 antibodies. DNA was stained with DAPI (blue).

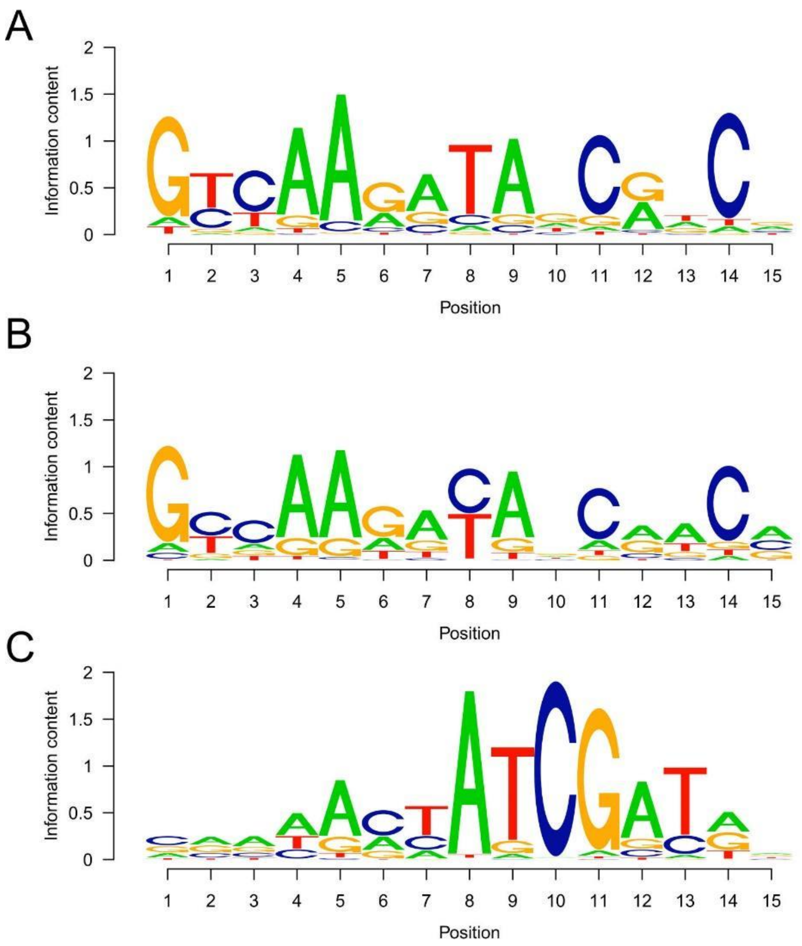

Figure S9. Consensus binding sites discovered by ChIPMunk for the top 100 (A), top 200 (B), and top 500 (C) peaks from the combined set of Mzfp1 binding sites obtained with rat and rabbit antibodies.

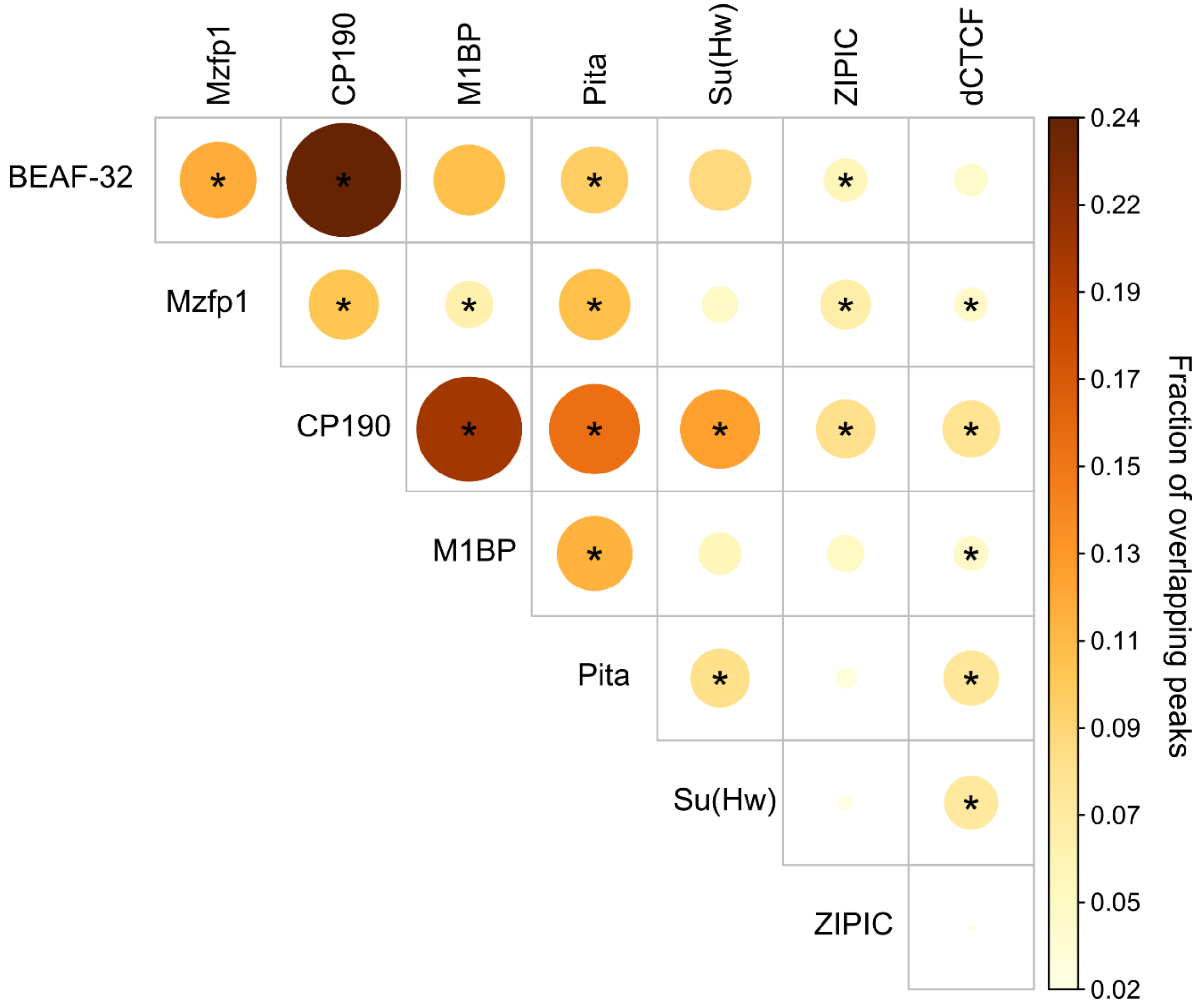

Figure S10. Colocalization analysis of protein pairs in housekeeping gene promoters. The size and color of the circles corresponds to the fraction of overlapping binding sites for each protein pair (from the union set of binding sites overlapping with the promoters of housekeeping genes). Asterisks denote significant colocalization according to Monte-Carlo simulations (see Methods).

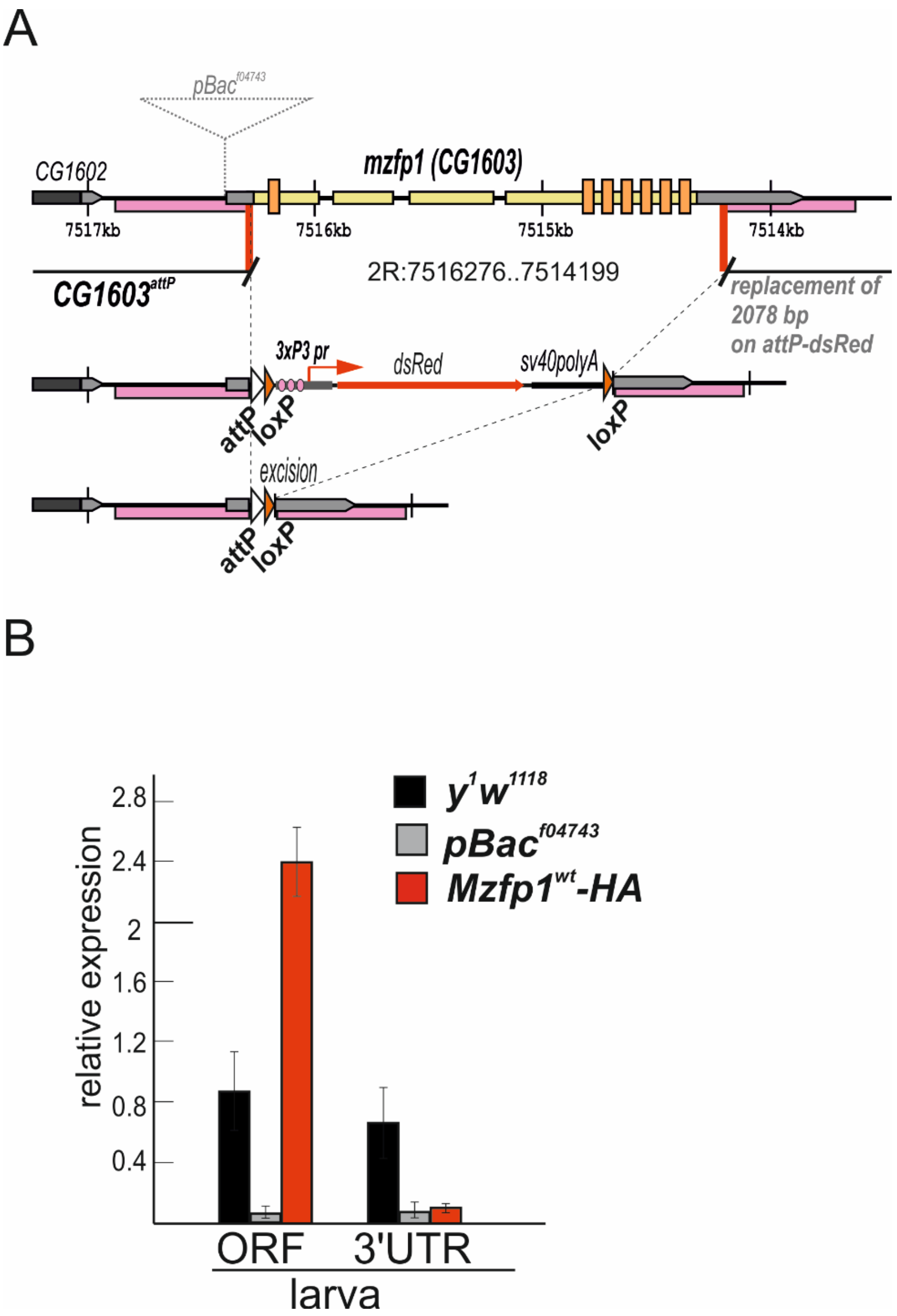

Figure S11. (A) CRISPR/Cas9 editing of the *CG1603* gene. The *CG1603* gene is shown by the yellow (coding region) and grey (*5'UTR* and *3'UTR*) regions. The insertion site for the PBac{WH} transgene in the *CG1603*^{f04743} mutation is indicated by the dotted triangle. CRISPR targets are shown as vertical red bars. The proximal and distal endpoints of the *CG1603*^{attP} deletion are indicated by breaks in the black line. The *dsRed* reporter (orange arrow), controlled by the *3P3* promoter, was used for the selection of the *CG1603* deletions. The *attP* and *loxP* sites were used for genome manipulations and are shown as white and orange arrows, respectively. **(B)** Expression levels of the *CG1603* gene in third-instar larvae in the wt (*y*^{1}*w*^{1118}), *CG1603*$^{-}$ *(pBac*^{f04743}*)*, and with overexpression of the *CG1603* gene in transgenic *U:Mzfp1*^{wt}*-HA* flies background. Individual transcript levels were determined by RT-qPCR with corresponding primers ('ORF' – primers from *CG1603* coding region; '3'UTR' – primers from *3'UTR* from *CG1603*) normalized relative to *RpL32* for the amount of input cDNA. The error bars show standard deviations of triplicate measurements.

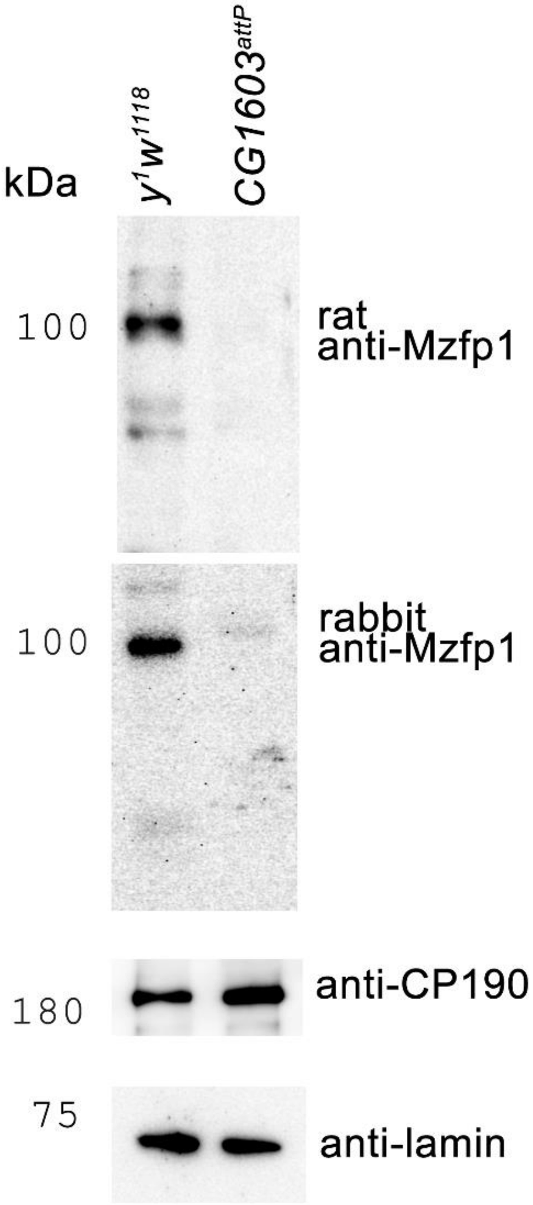

Figure S12. Immunoblot analysis (6% SDS PAGE) of protein extracts prepared from larvae of the y^1w^{1118} line and the y^1w^{1118}; $CG1603^{attP}/CG1603^{attP}$ line with deletion of the *mzfp1* gene with rat and rabbit antibodies against Mzfp1. Antibodies for lamin Dm0 and CP190 were used as internal controls.

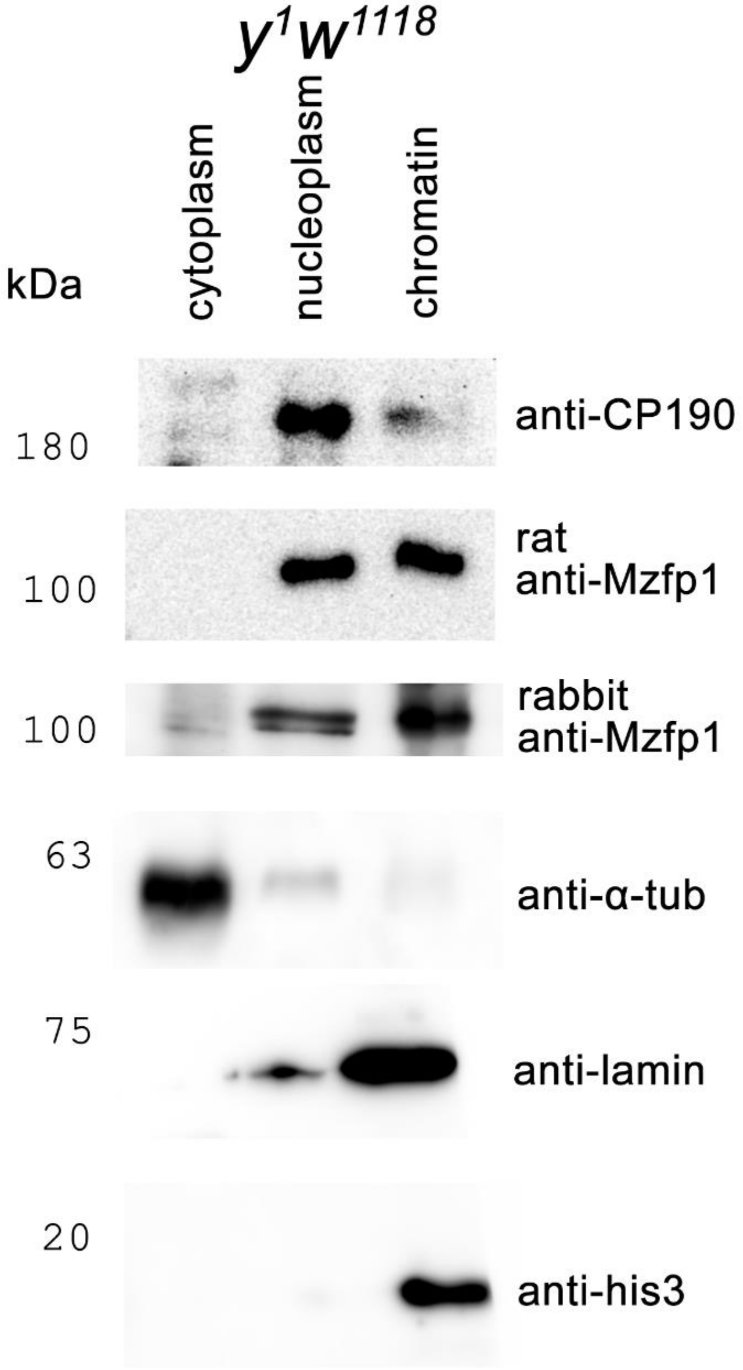

Figure S13. Immunoblot analysis (4-15% SDS PAGE) of cytoplasm, nucleoplasm, chromatin fractions prepared from two-day-old adult males of y^1w^{1118} line. Blots were stained with rat and rabbit antibodies against Mzfp1, antibodies against CP190 and control antibodies against α-tubulin (cytoplasmic marker), lamin Dm0 (nuclear marker), and histone H3 (his3, chromatin marker).

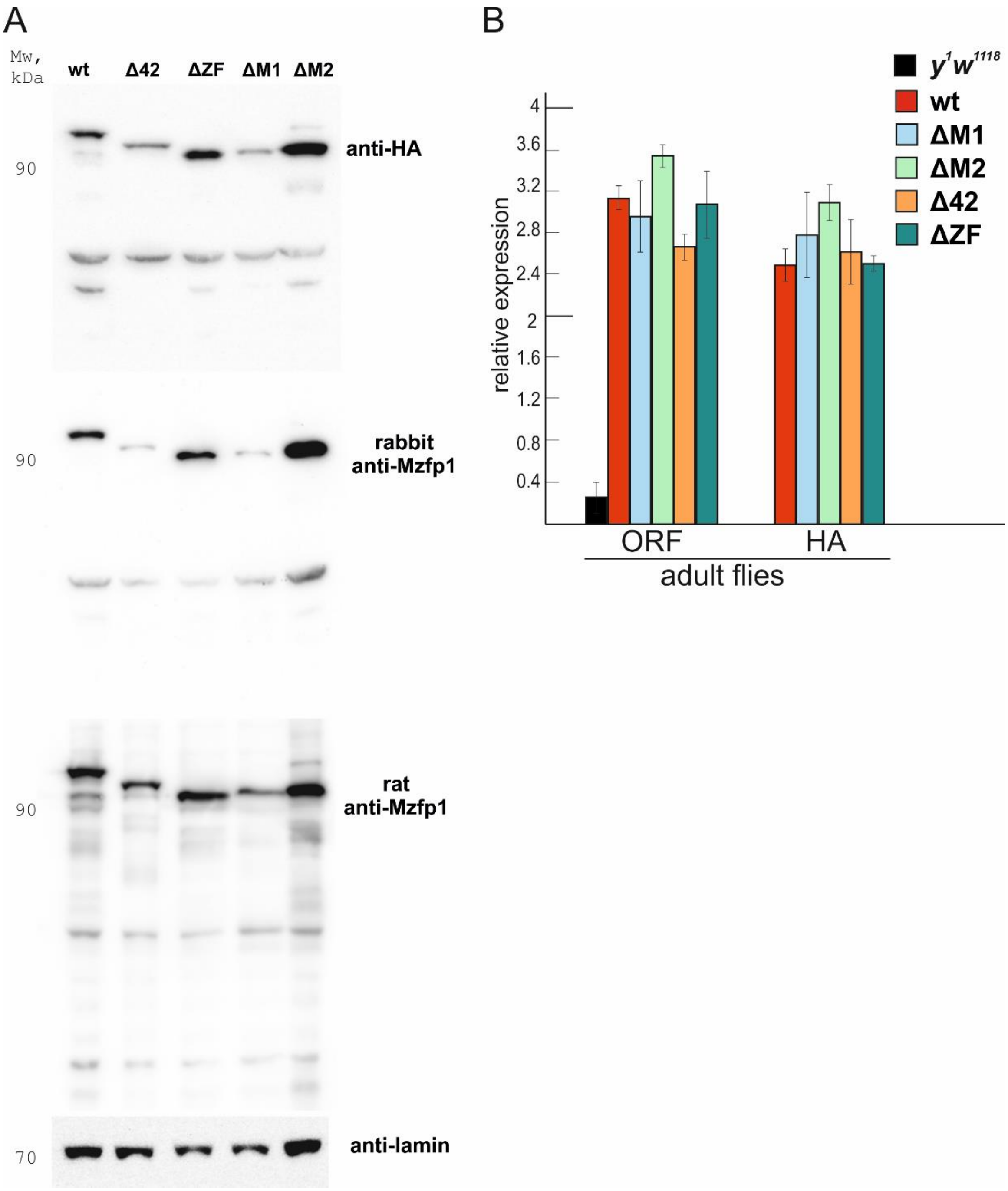

Figure S14. Analysis of the expression of Mzfp1 and its deletion derivatives. **(A)** Immunoblot analysis (8% SDS-PAGE) of protein extracts from transgenic flies expressing wild-type and deletion variants of Mzfp1 in adult flies: wt (Mzfp1^{wt}-HA), Δ42 (Mzfp1$^{\Delta42}$-HA), ΔZF (Mzfp1 $^{\Delta ZF}$-HA), Δ M1 (Mzfp $^{\Delta M1}$-HA), ΔM2 (Mzfp$^{\Delta M2}$-HA). Immunoblots were detected with antibodies against Mzfp1 (rat and rabbit), HA-epitope, and lamin Dm0. **(B)** Expression levels of the *CG1603* gene in adult transgenic flies expressing wild-type and deletion variants of Mzfp1. The y^1w^{1118} – native level of *CG1603* transcription in adult flies is shown. Individual transcript levels were determined by RT-qPCR with corresponding primers ('ORF' – primers from *CG1603* coding region; 'HA' – primers from HA-epitope) normalized relative to *RpL32* for the amount of input cDNA. Error bars show the standard deviation of triplicate measurements.

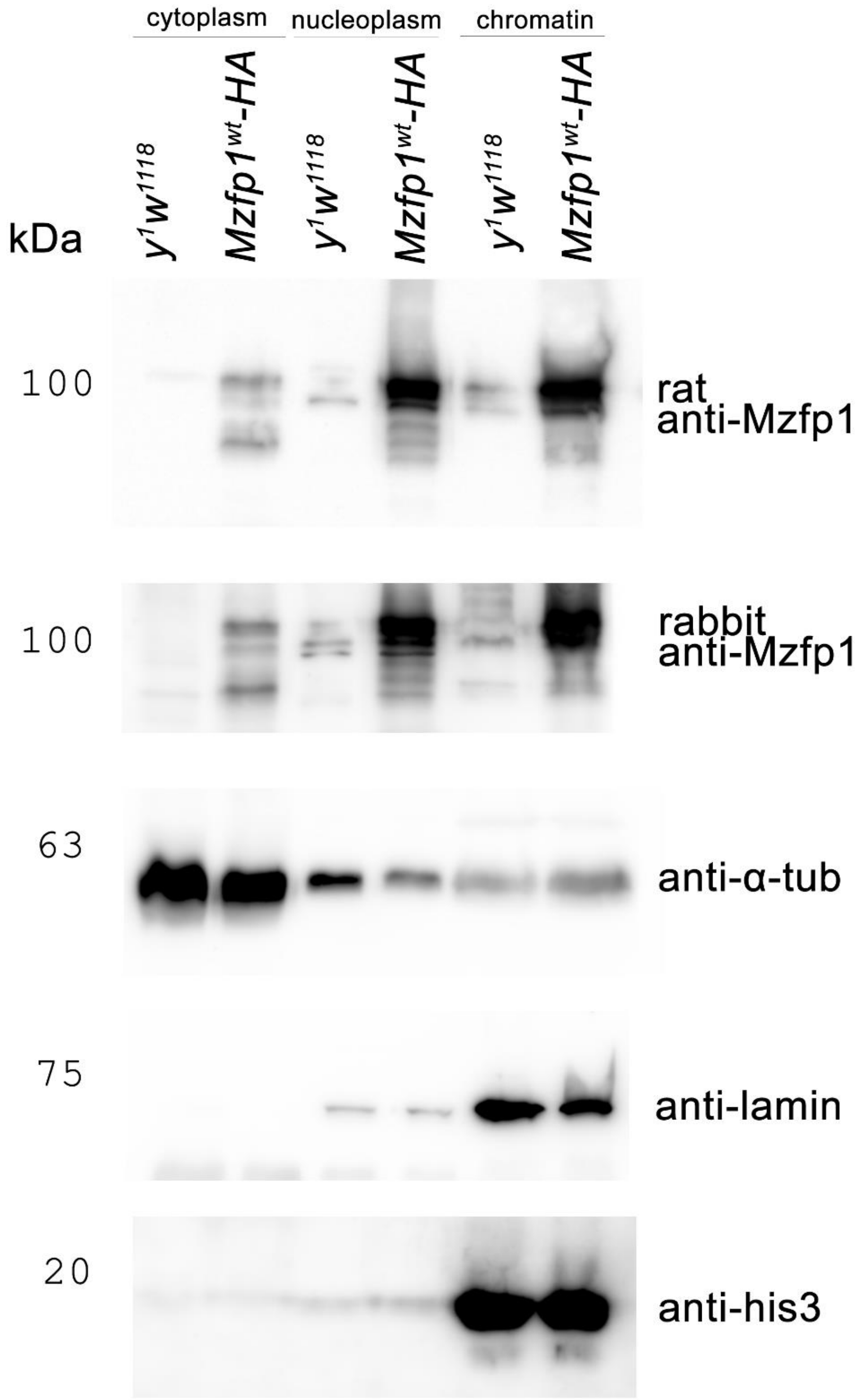

Figure S15. Immunoblot analysis (4-15% SDS PAGE) of cytoplasm, nucleoplasm, chromatin fractions prepared from two-day-old adult males of *y^1w^{1118}* and *$CG1603^{attP}/CG1603^{attP}$; $U{:}Mzfp1^{wt}/TM6,Tb$* (*$Mzfp1^{wt}$-HA*) lines. Blots were stained with rat and rabbit antibodies against Mzfp1 and control antibodies against α-tubulin (cytoplasmic marker), lamin Dm0 (nuclear marker), and histone H3 (his3, chromatin marker).

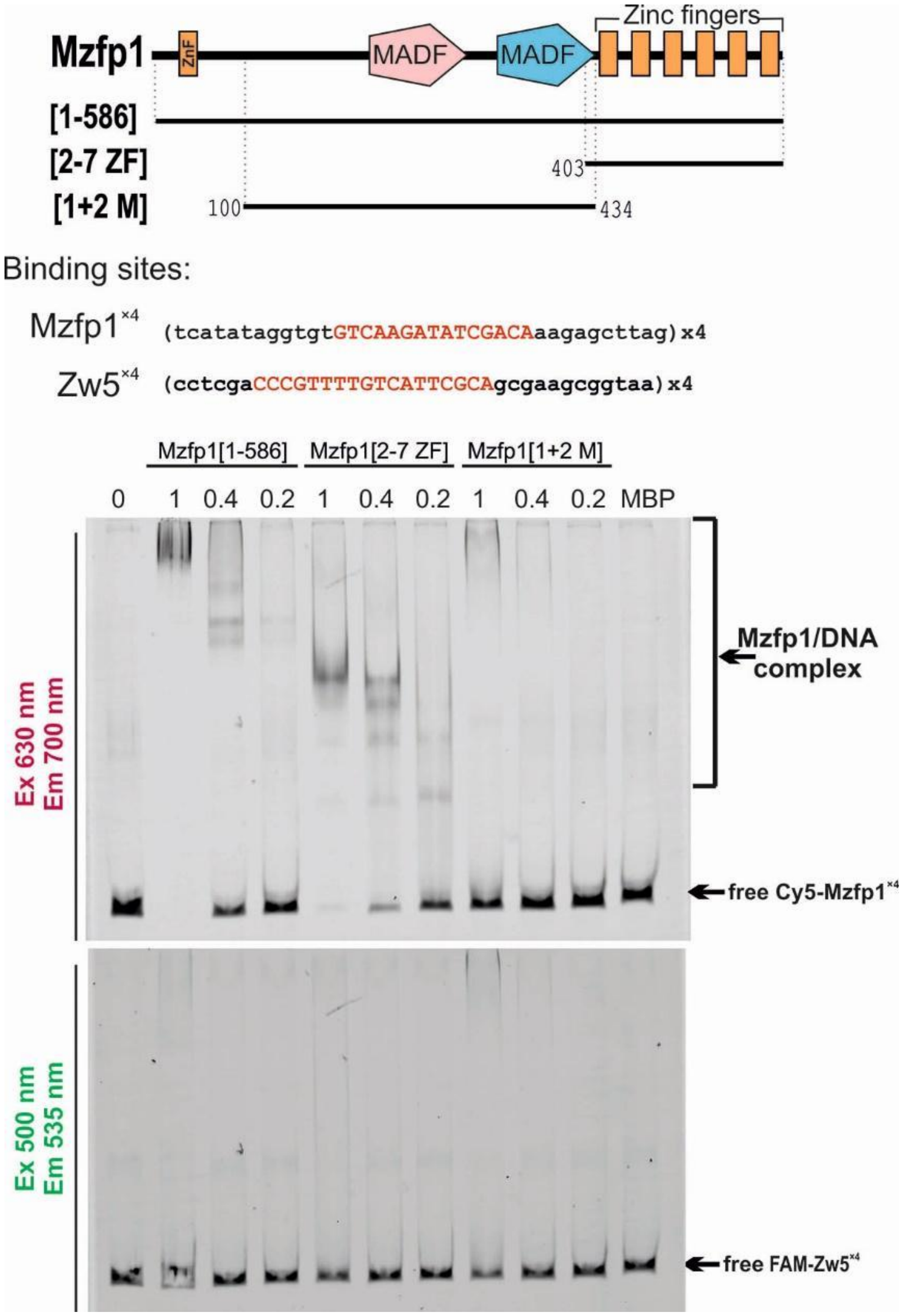

Figure S16. *In vitro* binding of full-sized Mzfp1, its zinc-finger C2H2 cluster (2-7 ZF), and two MADF domains with an artificial DNA fragment consisting of four binding sites for Mzfp1 (Mzfp1^{x4}), or for the architectural protein Zw5 (Zw5^{x4})(used as a negative control). Recombinant Mzfp1 fused with MBP or MBP alone were incubated with Mzfp1^{x4}, labeled with Cy5, and Zw5^{x4}, and labeled with FAM. Signals were detected for FAM-labeled fragments at an excitation wavelength of 500 nm and an emission wavelength of 535 nm and for the Cy5-labeled fragment at an excitation wavelength of 630 nm and an emission wavelength of 700 nm.

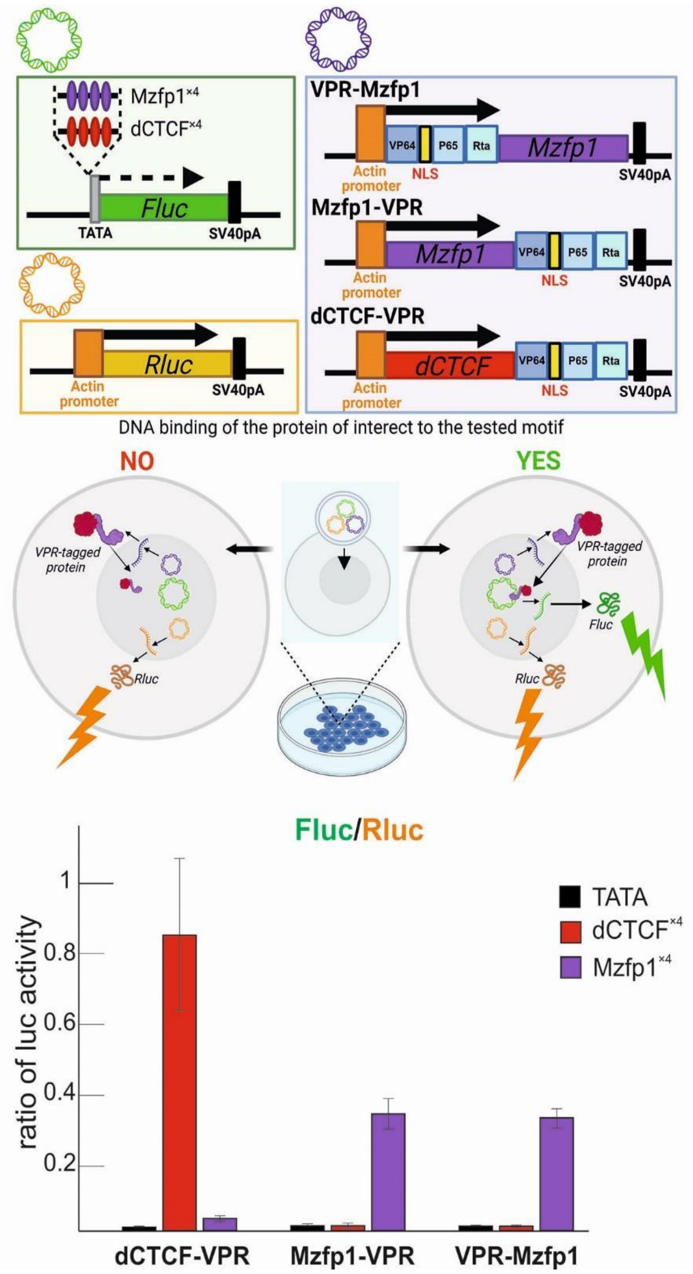

Figure S17. Analysis of Mzfp1 binding to its motif *in vivo*. Top: Schematic drawing of the reporter constructs employed: firefly luciferase reporter (TATA – Firefly luciferase with a minimal TATA box promoter; Mzfp1^{x4} – four motifs for Mzfp1 inserted above the TATA sequence; dCTCF^{x4}– four motifs for dCTCF inserted above the TATA sequence); Mzfp1- or dCTCF-expressing (the ORFs of dCTCF and Mzfp1 were fused with a VPR tag coding three strong activators under the actin 5C promoter); Renilla luciferase under the control of the actin 5C promoter (used to correct for variation in transfection efficiency). **Middle:** Scheme of the experiment. S2 cells were co-transfected with three plasmids. If the tested DNA-binding protein was able to bind with the motif inserted above the TATA sequence in the firefly luciferase reporter plasmid, the luminescence signal of firefly luciferase was detected. If the tested DNA-binding protein did not bind with the motif inserted above the TATA in the firefly luciferase reporter plasmid, the luminescence signal of firefly luciferase was absent. **Bottom:** Histogram showing the ratio of Firefly to Renilla luciferases in protein extracts from transfected S2 cells in the dual-luciferase assay. Each transfection experiment was performed in three independent biological replicates, and each lysate was measured in four technical replicates. Error bars show standard deviations of measurements of all summarized replicates. Created with BioRender.com.

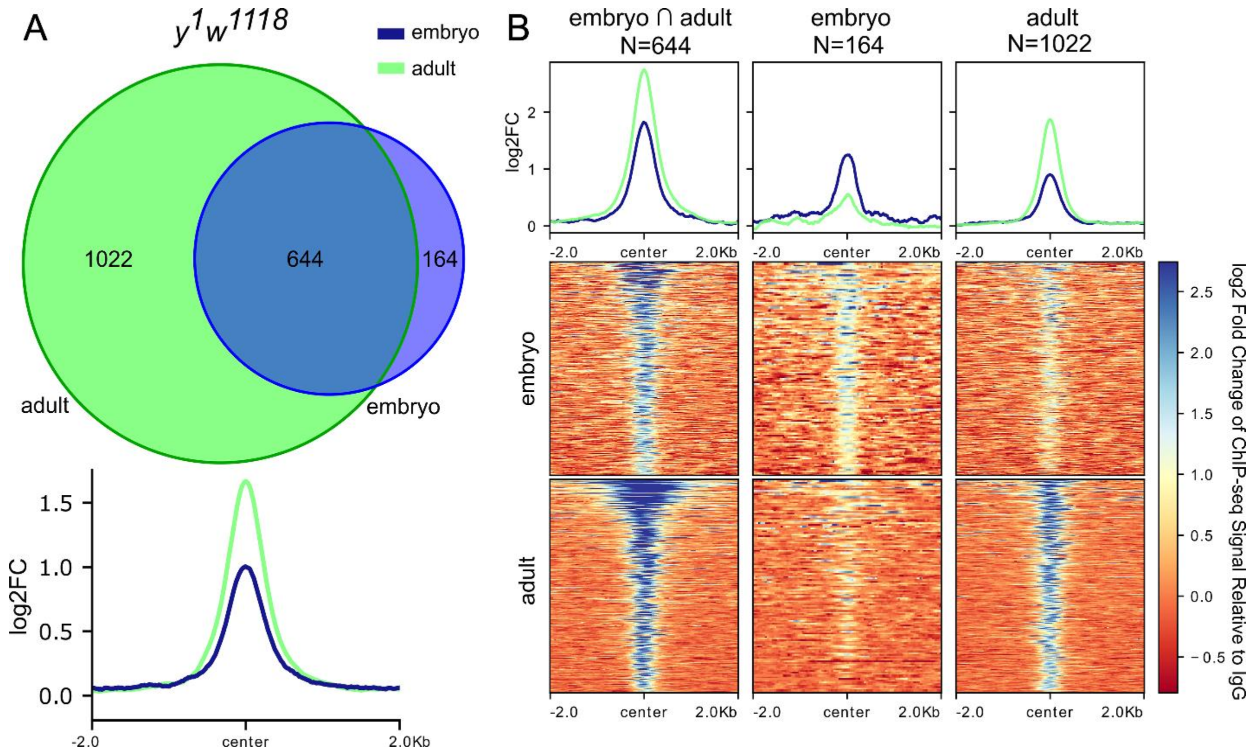

Figure S18. Colocalization of Mzfp1 binding sites in embryos and adults of the y^1w^{1118} line. **(A)** Number of overlapping and unique binding sites between embryos and adult flies. **(B)** Average ChIP-Seq signals (on the top) and signal heatmaps (on the bottom) for Mzfp1 in overlapping and unique binding sites between embryos and adult flies.

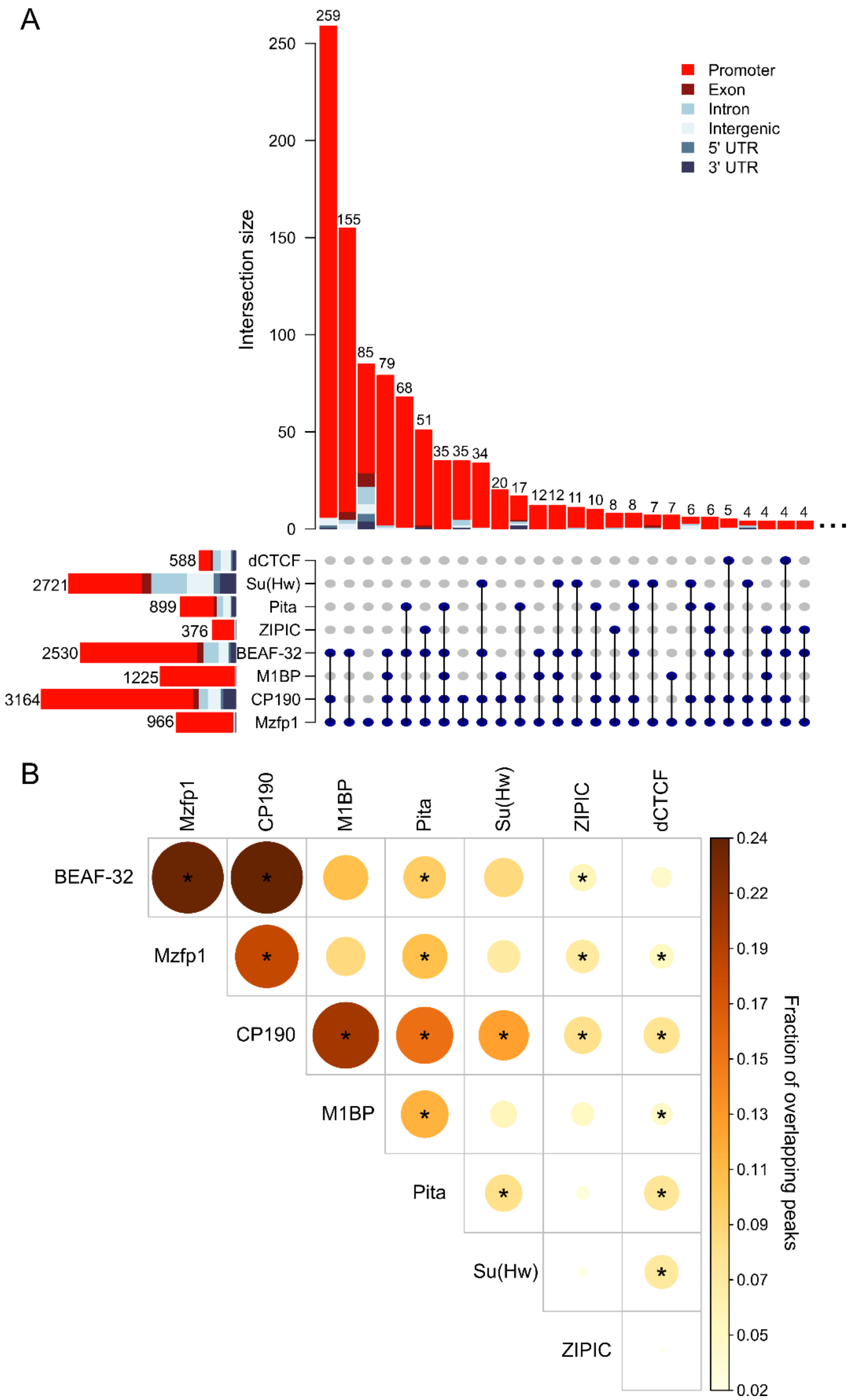

Figure S19. Colocalization of Mzfp1 with other proteins concerning a combined set of Mzfp1 binding sites obtained in y^1w^{1118} adult flies and embryos. **(A)** Colocalization of Mzfp1 binding sites with the binding sites for the M1BP, dCTCF, Su(Hw), Pita, ZIPIC, BEAF-32 and CP190 proteins. Only binding regions with motifs for all proteins except CP190 were considered in this analysis. **(B)** Analysis of protein pairs colocalization in the promoters of the housekeeping genes. The size and color of the circles corresponds to the fraction of overlapping binding sites for each protein pair (from the union set of binding sites overlapping with the promoters of housekeeping genes). Asterisks denote significant colocalization according to Monte-Carlo simulations (see Methods).

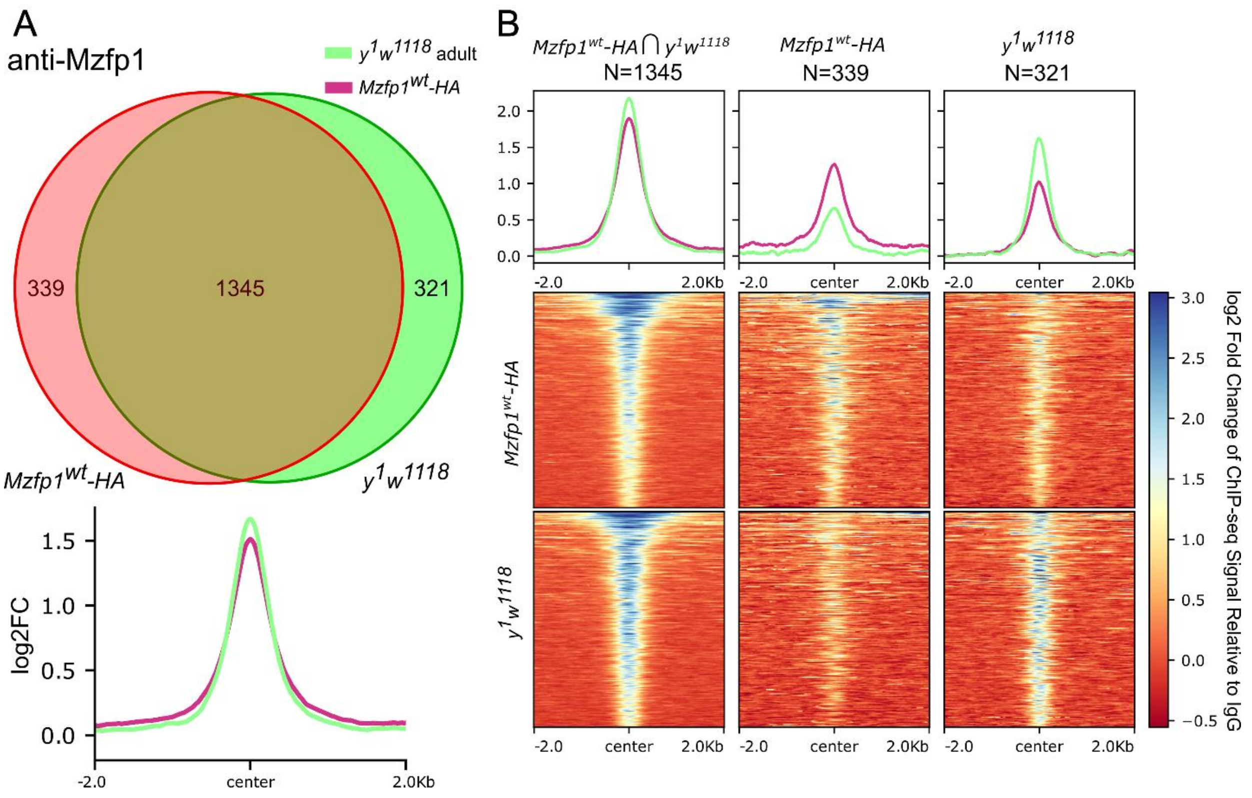

Figure S20. Colocalization of Mzfp1 binding sites in *y^{1}w^{1118}* and *Mzfp1^{wt}-HA* (*CG1603^{attP}*/*CyO; U:Mzfp1^{wt}*/*TM6,Tb*) lines from adult flies with anti-Mzfp1 antibodies. **(A)** Number of overlapping and unique binding sites between *y^{1}w^{1118}* and *Mzfp1^{wt}-HA* lines. **(B)** Average ChIP-Seq signals (on the top) and signal heatmaps (on the bottom) for Mzfp1 in overlapping and unique binding sites between *y^{1}w^{1118}* and *Mzfp1^{wt}-HA* lines.

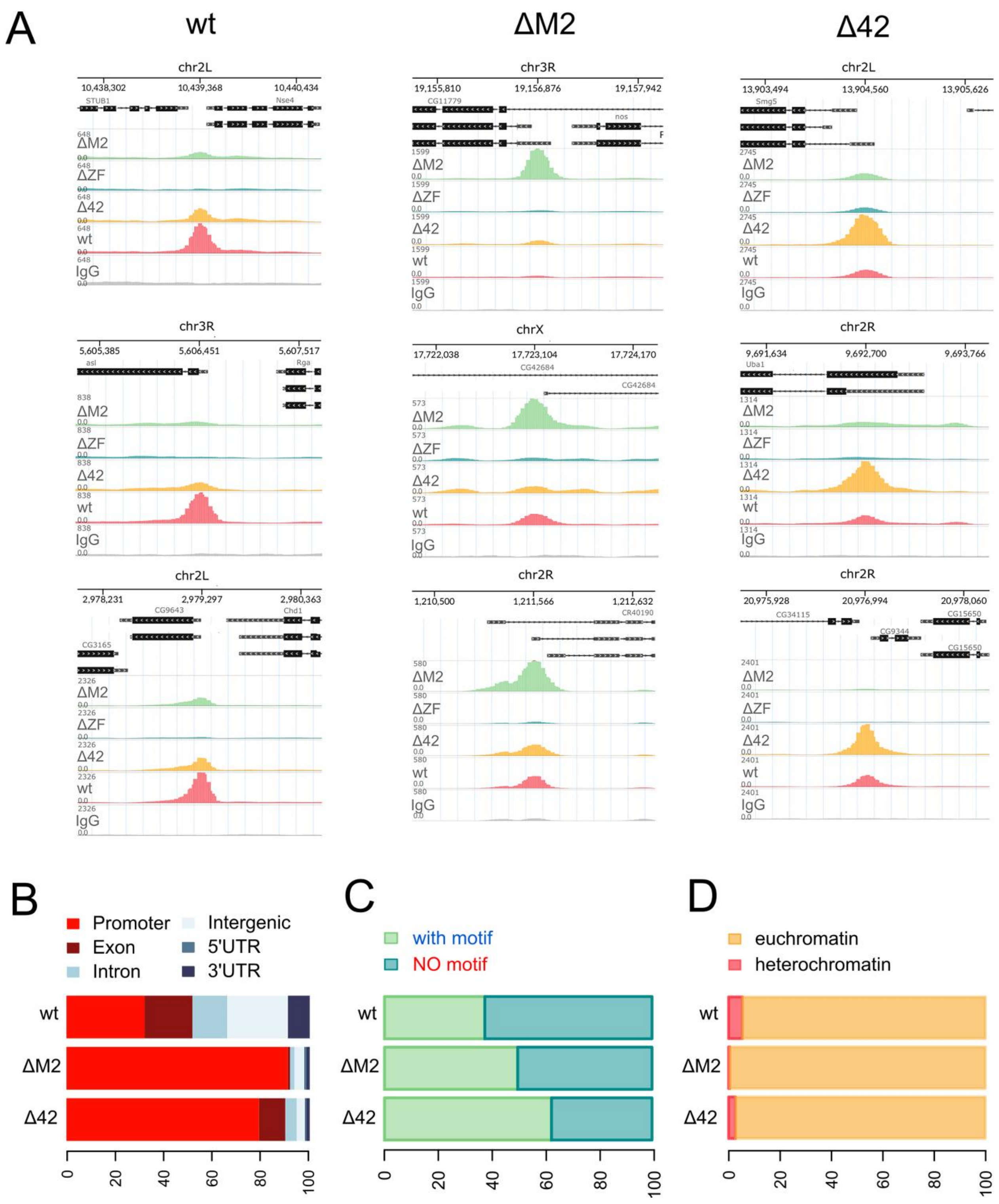

Figure S21. Characterization of Mzfp1 binding sites unique for $Mzfp1^{wt}$*-HA* (wt), $Mzfp1^{\Delta M2}$*-HA* (ΔM2) and $Mzfp1^{\Delta 42}$*-HA* (Δ42) lines with anti-HA antibodies (only binding sites intersecting with ones identified in $Mzfp1^{wt}$*-HA* anti-HA lines, $y^{1}w^{118}$ adults or embryos were considered in this analysis.). **(A)** Examples of binding profiles for unique Mzfp1 binding sites in wt, ΔM2 and Δ42 lines. **(B)** The distribution by genomic elements for sets of Mzfp1 binding sites unique for wt, ΔM2 and Δ42 lines. **(C)** The proportion of sites with the Mzfp1 motif for sets of Mzfp1 binding sites unique for wt, ΔM2 and Δ42 lines. **(D)** The distribution by euchromatic and heterochromatic regions for sets of Mzfp1 binding sites unique for wt, ΔM2 and Δ42 lines.

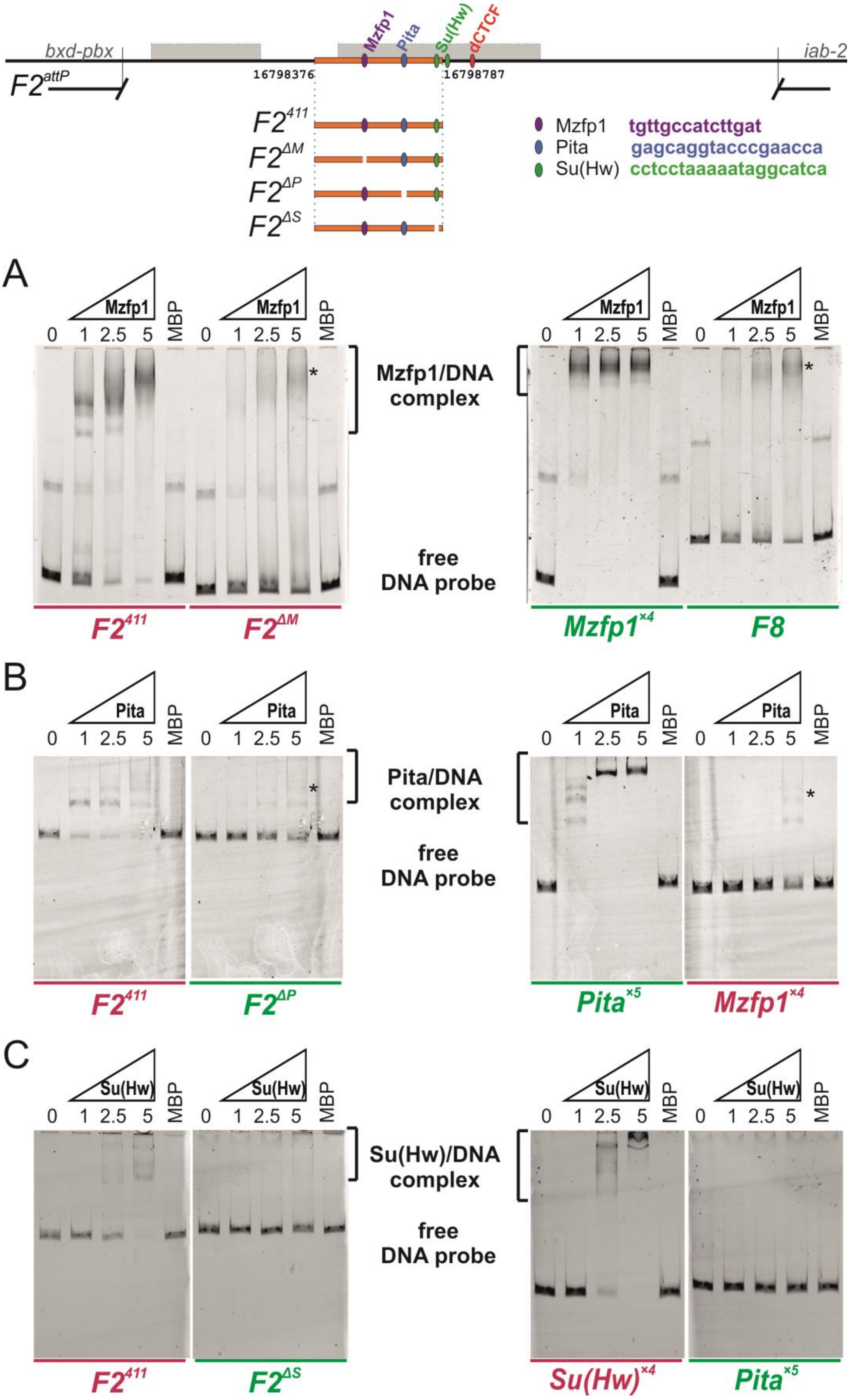

Figure S22. *In vitro* binding of Mzfp1, Pita, and Su(Hw) to $F2^{411}$ and its derivatives. The Mzfp1, Pita, and Su(Hw) motifs are shown as purple, blue, and green ovals with corresponding sequences from the $F2^{411}$ region on the top. An electrophoretic mobility shift assay was performed for binding of **(A)** Mzfp1, **(B)** Pita, and **(C)** Su(Hw) recombinant proteins fused with MBP or MBP alone with $F2^{411}$, its derivatives

(*F2*$^{\Delta M}$, *F2*$^{\Delta P}$, *F2*$^{\Delta S}$), and the controls (positive and negative) DNA fragments labeled with Cy5 (marked as red) and FAM (marked as green). Pita$^{\times 5}$, Su$^{\times 4}$, Mfzp$^{\times 4}$ and F8 (corresponding to Fab-8^{337}) sequences are listed in Supplementary table S1. Fab-8 is one of the BX-C boundary (1), that lacks Mfzp1 binding according to ChIP-seq data. Signals were detected for FAM-labeled fragments at an excitation wavelength of 500 nm and an emission wavelength of 535 nm and for Cy5-labeled fragment at an excitation wavelength of 630 nm and an emission wavelength of 700 nm. The asterisk indicates nonspecific binding caused by excess concentration of the recombinant protein, which begins to form aggregates.

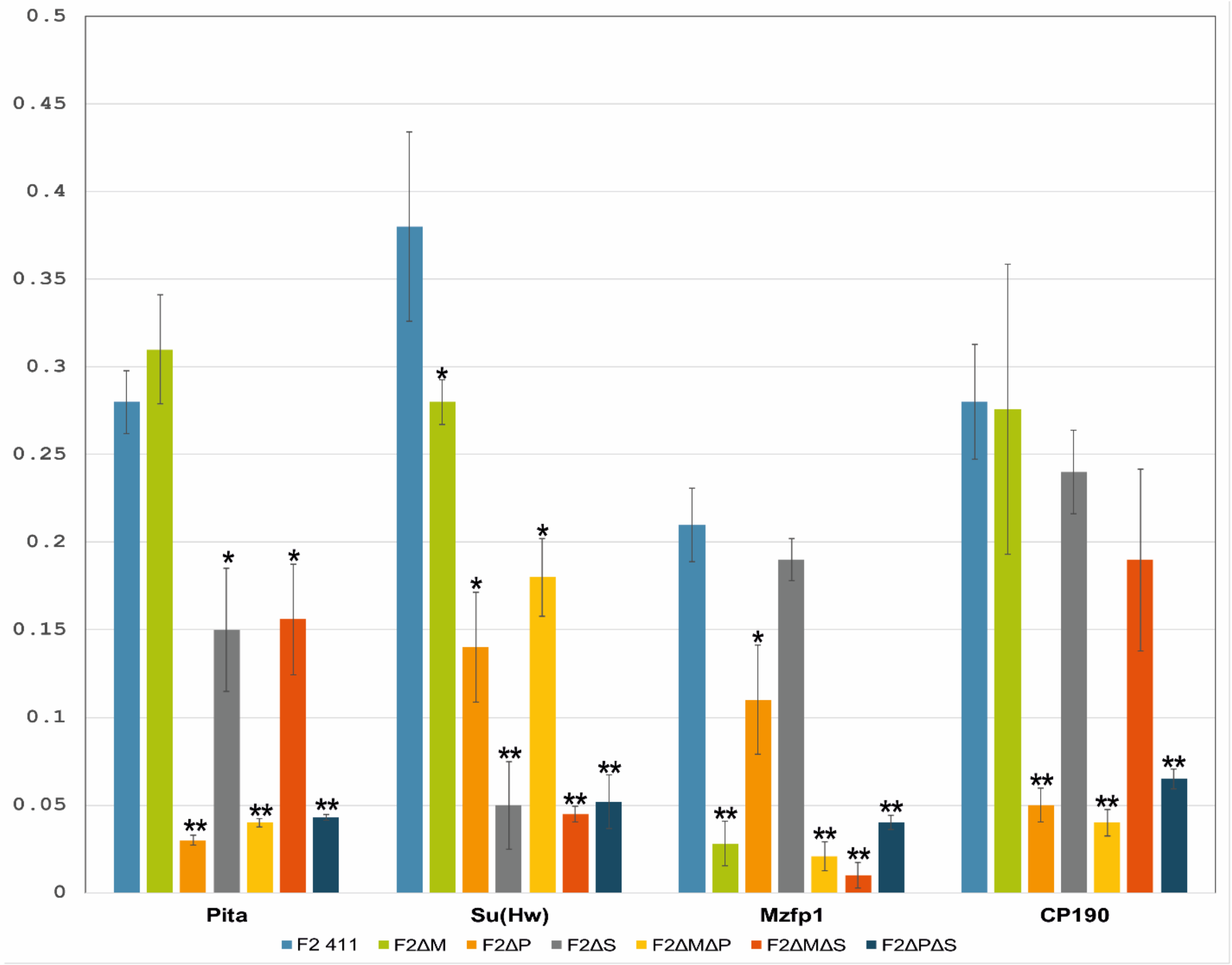

Figure S23. Binding of Pita, Su(Hw), Mzfp1, and CP190 with $F2^{411}$, $F2^{\Delta M}$, $F2^{\Delta P}$, $F2^{\Delta S}$, $F2^{\Delta M \Delta P}$, $F2^{\Delta M \Delta S}$, and $F2^{\Delta P \Delta S}$ elements. The results of ChIPs are presented as the percentage of input DNA, normalized against a positive genomic site: *62D*, for Su(Hw) binding; *50E*, for Pita; *94C*, for Mzfp1 and CP190 binding. The error bars indicate SDs of quadruplicate PCR measurements from three independent biological samples of chromatin. Asterisks indicate significance levels: $*P < 0.05$ and $**P < 0.01$.

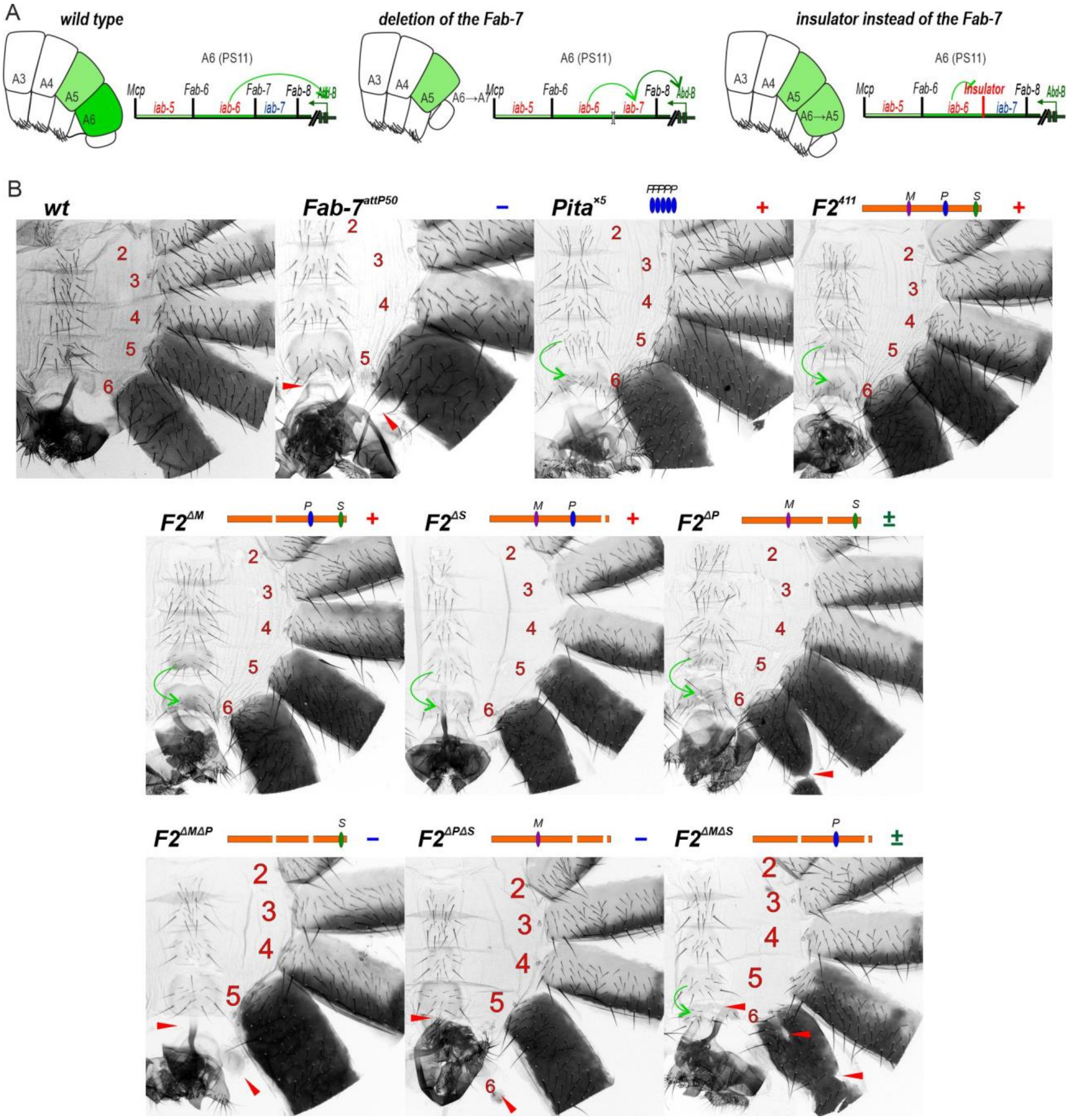

Figure S24. Analysis of the functional roles of Mzfp1, Su(Hw), and Pita in the activity of $F2^{411}$ inserted instead of the *Fab-7* boundary. **(A)** Schematic representation of an Fab-7^{attP50} deletion on the left and insertion of an insulator instead of an Fab-7 boundary on the right. Part of the *Abd-B* regulatory region (*iab-5* – *iab-7*) is presented. The horizontal green arrows show the *m*-transcript of *Abd-B*. The boundaries (*Mcp*, *Fab-6*, tested insulators inserted instead of *Fab-7*, and *Fab-8*) are indicated as vertical black bars. In the *Fab-7^{attP50}* deletion, the 1950 bp *Fab-7* boundary region was deleted and replaced with an *attP* site (2). **(B)** Morphology of the male abdominal segments (numbered) in the *Fab-7* replacement flies. The red arrows show the characteristic features of the morphology of the A6 segment, demonstrating the loss of insulator function. In *Pita^{x5}* (a strong insulator consisting of five Pita binding sites) adult males, the tergites of the A5 and A6 segments have the same rectangular shape and are pigmented, while A7 does not contribute to any visible cuticle structures. In *Fab-7^{attP50}* males (absence of an insulator between the *iab-6* and *iab-7* domains) the A6 segment is absent due to the A6→A7 transformation. The insertion of $F2^{411}$ into *Fab-7^{attP50}* restores the boundary function: adult males have A6 segments similar to A5. Similar results (normal insulator function) were obtained with $F2^{\Delta M}$ and $F2^{\Delta S}$. $F2^{\Delta P}$ did not completely restore insulator function: about 50% of males show splitting of the A6 tergite, indicating

insulator dysfunction in some of the cells that form the A6 segment. In $F2^{\Delta M\Delta P}$and $F2^{\Delta P\Delta S}$ the insulator activity is almost completely lost; in males, the structures defining the A6 segment are almost completely absent (in some males only a rudimentary A6 tergite is present). In $F2^{\Delta M\Delta S}$ substitution, there was partial inactivation of the insulator function: in males, the size of the A6 segment is reduced compared to the A5 segment.

References

1. Kyrchanova,O., Wolle,D., Sabirov,M., Kurbidaeva,A., Aoki,T., Maksimenko,O., Kyrchanova,M., Georgiev,P. and Schedl,P. (2019) Distinct Elements Confer the Blocking and Bypass Functions of the Bithorax Fab-8 Boundary. *Genetics*, **213**, 865–876.
